# A targeted multi-*omics* approach reveals *paraoxonase-1* as a determinant of obesity-associated fatty liver disease

**DOI:** 10.1186/s13148-021-01142-1

**Published:** 2021-08-13

**Authors:** Sara Diels, Bart Cuypers, Asta Tvarijonaviciute, Bruno Derudas, Evelien Van Dijck, An Verrijken, Luc F. Van Gaal, Kris Laukens, Philippe Lefebvre, Jose J. Ceron, Sven Francque, Wim Vanden Berghe, Wim Van Hul

**Affiliations:** 1 Department of Medical Genetics, University of Antwerp, Prins Boudewijnlaan 43, 2650 Edegem, Antwerp, Belgium; 2 Molecular Parasitology Unit, Department of Biomedical Sciences, Institute of Tropical Medicine, Antwerp, Belgium; 3 Adrem Data Lab, Department of Computer Science, University of Antwerp, Antwerp, Belgium; 4 Interdisciplinary Laboratory of Clinical Analysis INTERLAB-UMU, University of Murcia, Murcia, Spain; 5 Inserm, CHU Lille, Pasteur Institute of Lille, University of Lille, Lille, France; 6 Department of Endocrinology, Diabetology and Metabolic Disease, Antwerp University Hospital, Antwerp, Belgium; 7 Laboratory of Experimental Medicine and Pediatrics, University of Antwerp, Antwerp, Belgium; 8 Department of Gastroenterology and Hepatology, Antwerp University Hospital, Antwerp, Belgium; 9 Department of Biomedical Sciences, University of Antwerp, Antwerp, Belgium

**Keywords:** PON1, NAFLD, Obesity, Genetics, Methylation, -Omics, Integrative analysis

## Abstract

**Background:**

The multifactorial nature of non-alcoholic fatty liver disease cannot be explained solely by genetic factors. Recent evidence revealed that DNA methylation changes take place at proximal promoters within susceptibility genes. This emphasizes the need for integrating multiple data types to provide a better understanding of the disease’s pathogenesis. One such candidate gene is *paraoxonase-1* (*PON1*). Substantial interindividual differences in PON1 are apparent and could influence disease risk later in life. The aim of this study was therefore to determine the different regulatory aspects of PON1 variability and to examine them in relation to the predisposition to obesity-associated fatty liver disease.

**Results:**

A targeted multi-*omics* approach was applied to investigate the interplay between *PON1* genetic variants, promoter methylation, expression profile and enzymatic activity in an adult patient cohort with extensive metabolic and hepatic characterisation including liver biopsy. Alterations in PON1 status were shown to correlate with waist-to-hip ratio and relevant features of liver pathology. Particularly, the regulatory polymorphism rs705379:C > T was strongly associated with more severe liver disease. Multivariable data analysis furthermore indicated a significant association of combined genetic and epigenetic *PON1* regulation. This identified relationship postulates a role for DNA methylation as a mediator between *PON1* genetics and expression, which is believed to further influence liver disease progression via modifications in PON1 catalytic efficiency.

**Conclusions:**

Our findings demonstrate that vertical data-integration of genetic and epigenetic regulatory mechanisms generated a more in-depth understanding of the molecular basis underlying the development of obesity-associated fatty liver disease. We gained novel insights into how NAFLD classification and outcome are orchestrated, which could not have been obtained by exclusively considering genetic variation.

**Supplementary Information:**

The online version contains supplementary material available at 10.1186/s13148-021-01142-1.

## Background

Non-alcoholic fatty liver disease (NAFLD), characterized by significant lipid deposition in hepatocytes, is set to become the most common liver disease worldwide [1]. While clinical, demographic and environmental factors are implicated in the development of NAFLD, obesity has been recognized as one of the main risk factors [2]. The disease affects 25% of the world population on average and increases up to 65–90% in patients with obesity [3, 4]. These high prevalence rates indicate NAFLD as an important health concern and emphasize the need for disease management. Moreover, the potential transition of isolated steatosis (i.e. NAFL) to more severe non-alcoholic steatohepatitis (NASH), fibrosis and cirrhosis can be recognized as a major contributor to adverse health outcomes (e.g. heart disease, cancer, Covid-19) and mortality in the general population [5–7]. Although accurate prevention and treatment are clearly required, they are challenging as obesity and associated NAFLD are complex heterogeneous diseases for which the mechanisms that contribute to their onset and progression remain only partially elucidated.

The multifactorial nature of both disorders implicate the presence of dietary habits, physical activity, genetics, epigenetics and the environment as important aspects of its pathogenesis. Numerous studies have estimated that 40–70% of the interindividual variability in body mass index (BMI) can be attributed to genetic factors [8–14]. Given this estimated heritability, extensive genome-wide association studies have been performed to determine the influence of common and rare variants in the pathogenesis of complex metabolic disease. Even though a large number of obesity-associated loci were identified (> 500), the proportion of variance explained (16–40%) remains lower than the proposed heritable risk [15]. The same is true for NAFLD/NASH, for which several single nucleotide polymorphisms (SNPs) have been identified as genetic modifiers [16]. This indicates the need to explore other forms of genetic variation concerning complex hepatometabolic diseases. An important role must be attributed to the interaction between genetics and the environment. The “common disease genetic and epigenetic” hypothesis proposes that in addition to genetic variation, epigenetics provides an added layer of variation that mediates the gene-environment relationship [17, 18]. It has been demonstrated that there is a significant interplay between obesity-associated genetic variants and environmental factors (e.g. diet, endocrine disruptors, medication) with DNA methylation changes at proximal promoters and enhancers [19]. This highlights the importance of integrating genetic and epigenetic data to provide a more detailed understanding of the disease pathophysiology. Although epigenetic regulation of metabolic health also involves dynamic changes in histone modifications, chromatin accessibility, and RNA interference, most clinical diagnostic studies focus exclusively on associations between genetic versus DNA methylation changes [20]. In general DNA methylation changes are more robust than chromatin changes, which typically are transient and susceptible to a quick turnover [21]. Moreover, since most preserved clinical patient samples (liver biopsy material) contain limited amounts of gDN, clinical diagnostic epigenetic biomarker platforms for DNA methylation quantification are most suitable for sensitive and accurate detection of small quantitative DNA methylation changes at nucleotide resolution, rather than poor quantitative methods measuring regional chromatin changes [22].

Based on preliminary data from our lab and others, we hypothesize that paraoxonase-1 (PON1) is a potential determinant in the development of hepatometabolic disease. PON1 is a multifunctional enzyme synthesized in the liver and secreted in the plasma where it associates with high-density-lipoproteins (HDL). It hydrolyses a wide variety of substrates (e.g. lactones, organophosphorus pesticides, arylesters) and is best known for its antioxidant and anti-inflammatory properties by detoxification of low-density-lipoproteins (LDL) and HDL metabolites [23–26]. Recent advances from association, (epi)genetic and animal studies further emphasize a protective role of PON1 against environmental exposure, obesity and NAFLD. A previous study by our research group identified remarkable associations between the *PON1* genotype and adverse epigenetic marks in endocrine pathways related to childhood obesity and high body fat content at school age [27]. More particularly, we found that (1) T-allele carriers of the regulatory variant rs705379:C > T have higher DNA-methylation values at the *PON1* promoter region, negatively affecting organophosphorus pesticide hydrolysis, and (2) the coding variant rs662:T > C influences methylation of genes in several relevant pathways involved in the regulation of appetite and type II diabetes mellitus signalling. Other studies have also associated reduced *PON1* expression with adverse lipid metabolism and recognized this as a risk factor for obesity, liver steatosis and its more severe subtype steatohepatitis [28–30]. This link has been confirmed by the observation that *pon1*-deficient mice, fed a high-fat high-cholesterol diet, develop severe steatosis in the liver [31]. Genetic studies in patients with obesity further indicate a possible correlation of the disease occurrence with *PON1* polymorphisms [28, 32]. However, many inconsistencies and unanswered questions remain.

Current *PON1* research in obesity and NAFLD focused on either genetic, epigenetic or functional data to explain its function in pathophysiology. None of these studies have combined these different elements to examine complex obesity phenotypes in relation to *PON1* polymorphisms, DNA methylation status and associated changes in gene expression and enzymatic activity. The availability of a hepatometabolic patient cohort, with extensive clinical data from different patient samples, enabled us to investigate PON1 variability in a larger context than previously considered. Therefore, the aim of our study was to integrate these different data types to evaluate to what extent PON1 status is associated with NAFLD presence and severity and with the associated metabolic dysregulation.

## Results

Variability within the different PON1 -*omics* levels was explored in a hepatometabolic patient cohort (HEPADIP cohort) to identify clinicopathological associations of PON1 regulation with predisposition to obesity-associated fatty liver disease. The complete study population consisted of adult patients (71.5% females, 28.5% males) with a weight-related problem, most of whom were diagnosed with obesity (95.8% of the overall cohort of 790 patients). For 329 participants (41.6%), none of the criteria to propose a liver biopsy were present and therefore no histological characteristics were available. In the other patients (*N*_liver biopsy_ = 461), at least one criterion was met or a liver biopsy was proposed as the patient underwent bariatric surgery. The distributions (*x*:*y*:*z*) according to the grade of steatosis, ballooning, lobular inflammation, adjusted SAF activity, the NAFLD Activity Score (NAS), and the stage of fibrosis were determined and showed an overrepresentation of NAFLD stage 3 patients “NASH + fibrosis grade 1” (45.6% of the liver biopsy cohort of 461 patients). The mean, standard error, minimum and maximum for all clinical and biochemical parameters as well as the absolute values for categorical variables, evaluated in this study, are listed in Table 1. Different subcohorts were present dependent on the PON1 level examined (Additional file 1: Fig. S1). The main characteristics for each of these subpopulations can be found in Additional file 1: Tables S1–S3.

**Table 1:** Study population characteristics

	Mean	(Minimum/maximum)
Age (years)	43 ± 0.45	(18–74)
Weight (kg)	110.7 ± 0.8	(65.8–226.6)
Height (m)	1.68 ± 0.003	(1.47–2.06)
BMI (kg/m^2^)	38.8 ± 0.22	(24.97–69.13)
Waist circumference (cm)	116.7 ± 0.5	(83.5–193)
Hip circumference (cm)	122.2 ± 0.4	(95–160)
Waist-to-hip ratio	0.96 ± 0.004	(0.66–1.35)
Fat free mass (kg)	56.2 ± 0.4	(36.5–110)
Fat mass (kg)	54.3 ± 0.5	(16.9–134.5)
Fat mass (%)	48.87 ± 0.27	(22.3–65.3)
Total abdominal adipose tissue (cm^2^)	799.5 ± 6.6	(246–1386)
Visceral abdominal tissue (cm^2^)	195.9 ± 3.2	(29–567)
Subcutaneous abdominal tissue (cm^2^)	603.6 ± 5.6	(166–1059)
Systolic blood pressure (mmHg)	127.1 ± 0.5	(90–180)
Diastolic blood pressure (mmHg)	75.45 ± 0.37	(49–115)
Creatinine kinase (mg/dL)	0.81 ± 0.006	(0.38–1.64)
Aspartate aminotransferase (U/L)	23.6 ± 0.5	(7–133)
Alanine aminotransferase (U/L)	35.8 ± 0.8	(7–265)
Alkaline phosphatase (U/L)	79.94 ± 0.79	(30–236.03)
gamma-glutamyltransferase (U/L)	43.43 ± 1.19	(11.79–315.16)
Total cholesterol (mg/dL)	199 ± 1.40	(75–400.44)
High-density-lipoprotein Cholesterol (mg/dL)	50.83 ± 0.49	(24–107.17)
Triglycerides (mg/dL)	152.97 ± 2.93	(17–823.01)
Low-density-lipoprotein cholesterol (mg/dL)	117.81 ± 1.24	(17.6–294.76)
Insulin resistance (HOMA)	4.27 ± 0.23	(0.07–147.65)
Steatosis (0:1:2:3)	109:151:119:82	
Ballooning (0:1:2)	139:187:135	
Lobular inflammation (0:1:2:3)	148:208:75:30	
Fibrosis stage (0:1:2:3:4)	287:91:51:28:3	
Adjusted SAF activity (0:1:2:3:4:5)	100:71:112:118:44:16	
NAFLD activity score (0:1:2:3:4:5:6:7:8)	75:44:48:62:84:60:50:30:8	
NAFLD staging (1:2:3:4)	61:80:210:53	

Patient clinical and biochemical variable statistics are represented for the complete HEPADIP cohort (*N*_total_ = 790; 565 females—225 males). Values are expressed as the mean ± standard error of the mean. Lower and upper limits are indicated as minimum/maximum. The distribution of patients (*x*:*y*:*z*) according to the histological characteristics (*N*_liver biopsy_ = 461) are shown as absolute values. The histological criteria were evaluated by the NASH-CRN scoring system

### Common polymorphisms and promoter methylation define PON1 status

#### Genotype-specific regulation of PON1 status

**Fig. 1:**
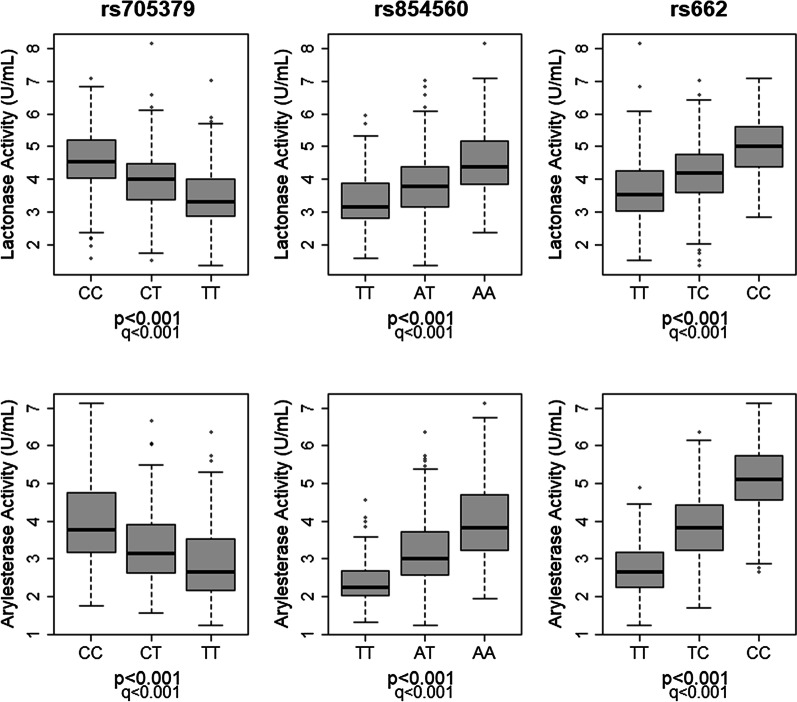
Relationship between *PON1* genetics and substrate-specific enzymatic activity. The different boxplots indicate the relationship between each genotype of the three common *PON1* polymorphisms rs705379:C > T, rs854560:A > T, rs662:T > C on serum lactonase (**a**) and arylesterase (**b**) in a population of patients with a wide range of (hepato)metabolic derangements. A total of 714 serum samples were analysed for which genotype distribution over the three PON1 variants is as follows: 26% CC, 46% CT and 28% TT for rs705379:C > T; 16% TT, 46% AT and 38% AA for rs854560:A > T; and 50% TT, 41% CT and 9% CC for rs662:T > C. Activity levels are expressed as units per millilitre of serum, in which 1 unit equals 1 mmol of 5-thiobutyl butyrolactone (lactone-hydrolysing activity) or phenyl acetate (arylester-hydrolysing activity) hydrolysed/min. The significance level (*p*) and FDR threshold (*q*) were set at 0.05

The complete HEPADIP cohort was genotyped to evaluate whether the three common polymorphisms rs705379:C > T (NM_000446.6;c.-108C > T), rs854560:A > T (NP_000437.3:p.Leu55Met) and rs662:T > C (NP_000437.3:p.Gln192Arg) were associated with *PON1* expression and PON1 lactonase and arylesterase activity. A strong correlation (*q* < 0.001; Additional file 1: Table S4) was present between the SNPs and both enzymatic activities. High activity was observed in patients carrying the C-allele of regulatory polymorphism rs705379 and in A-allele carriers and C-allele carriers of coding variants rs854560 and rs662, respectively (Fig. 1). The same genetic effect was found for *PON1* expression (Additional file 1: Fig. S2). However, a significant association could only be assigned to rs854560:A > T (*p* = 0.0304; EE = 0.18 change in methylation compared to A-allele carriers; STDE = 0.0757), which did not persist after multiple testing correction (*q* = 0.0912). The genetic effects on PON1 status were independent; i.e. none of the common polymorphisms were in linkage with each other [33]. The three *PON1* polymorphisms together explain 30.5% of lactone-hydrolysing activity and 58% of arylester-hydrolysing activity.

#### Methylation controls PON1 status in a location-dependent manner

The DNA methylation level for a total of 11 CpG sites was measured at the *PON1* promoter region. Comparison of methylation variability across the 11 CpG sites revealed a similar pattern between ten of the distinct CpGs for genetics, expression and activity (Additional file 1: Fig. S3–S5). Only CpG site -*108*, which is also known as the location of SNP rs705379:C > T, showed a deviating pattern. Accordingly, methylation parameters were considered as average promoter methylation (summed average of 10 CpG sites) and -*108* methylation. Overall, methylation level percentages varied between 6–60%; lower methylation levels were observed (CpG-*108*_mean_ = 10.4%) at CpG site *-108* in comparison to the average promoter methylation (CpG_mean_ = 27.9%).

**Fig. 2:**
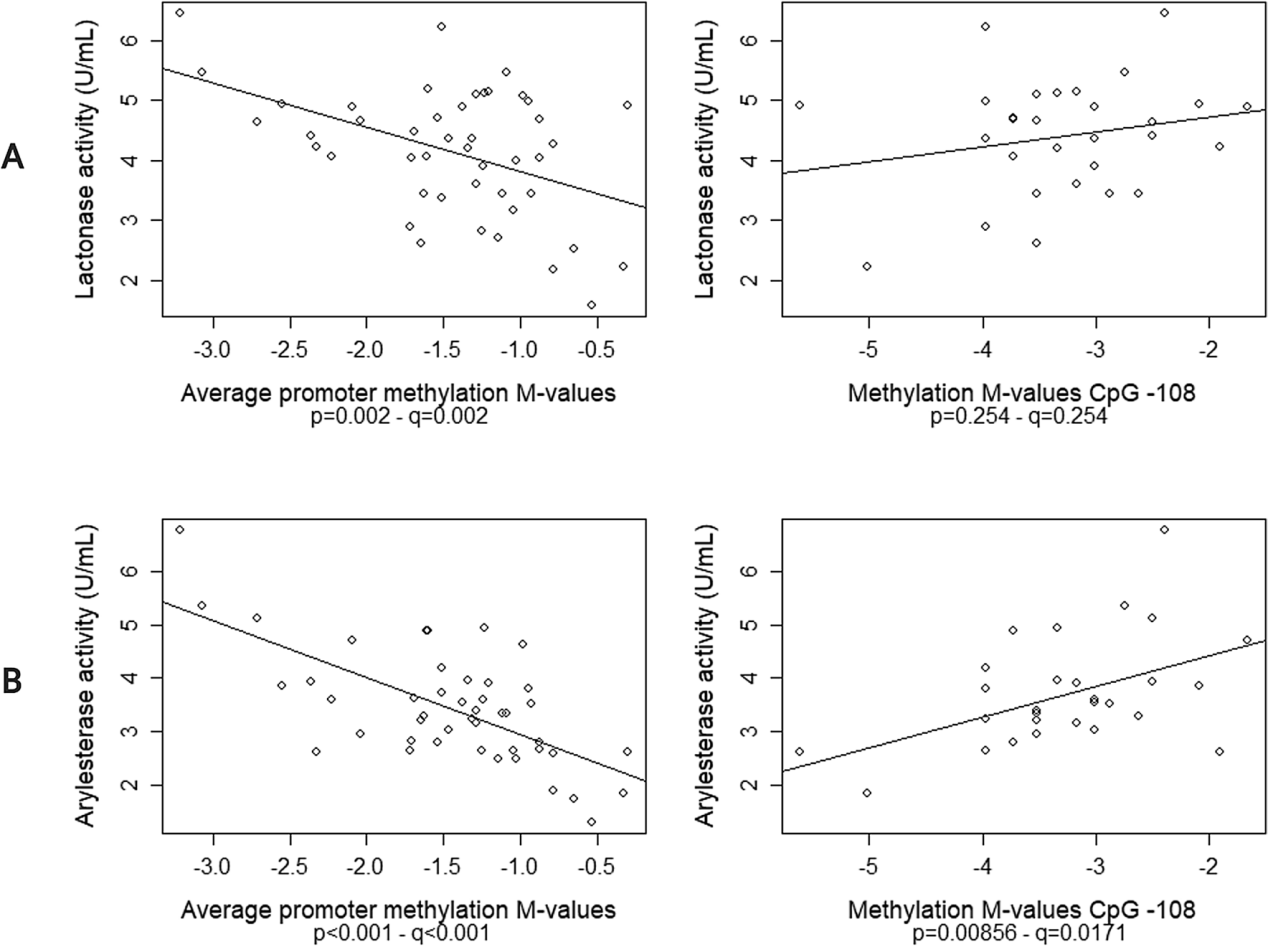
Relationship between *PON1* epigenetics and substrate-specific enzymatic activity. Graphs indicating the association of average promoter methylation and -*108* methylation on serum lactonase (**a**) and arylesterase (**b**) in a population of 45 patients with a wide range of (hepato)metabolic derangements. Methylation is represented as *M*-values; positive *M*-values mean that more molecules are methylated than unmethylated (> 50% methylation) while negative *M*-values mean the opposite (< 50% methylation). Activity levels are expressed as units per millilitre of serum, in which 1 unit equals 1 mmol of 5-thiobutyl butyrolactone (lactone-hydrolysing activity) or phenyl acetate (arylester-hydrolysing activity) hydrolysed/min. The significance level (*p*) and FDR threshold (*q*) were set at 0.05

The effect of epigenetics on PON1 status was assessed and demonstrated a significant association between average promoter hypermethylation and reduced expression (*p* = 0.01, EE = − 0.26 change in expression in relation to increased methylation; STDE = 0.09321; *q* = 0.02; Additional file 1: Fig. S6A). The opposite effect was found for -*108* methylation (hypomethylation results in decreased expression), although this was not significant (*p* = 0.143, EE = 0.16, STDE = 0.1045; *q* = 0.143; Additional file 1: Fig. S6B). The same pattern was equally present for both enzymatic activities; increased average promoter methylation led to decreased activity while increased -*108* methylation resulted in elevated activity levels (Fig. 2). All these correlations were significant, except for -*108* DNA methylation associated with lactone-hydrolysing activity (Additional file 2: Table S6).

**Fig. 3:**
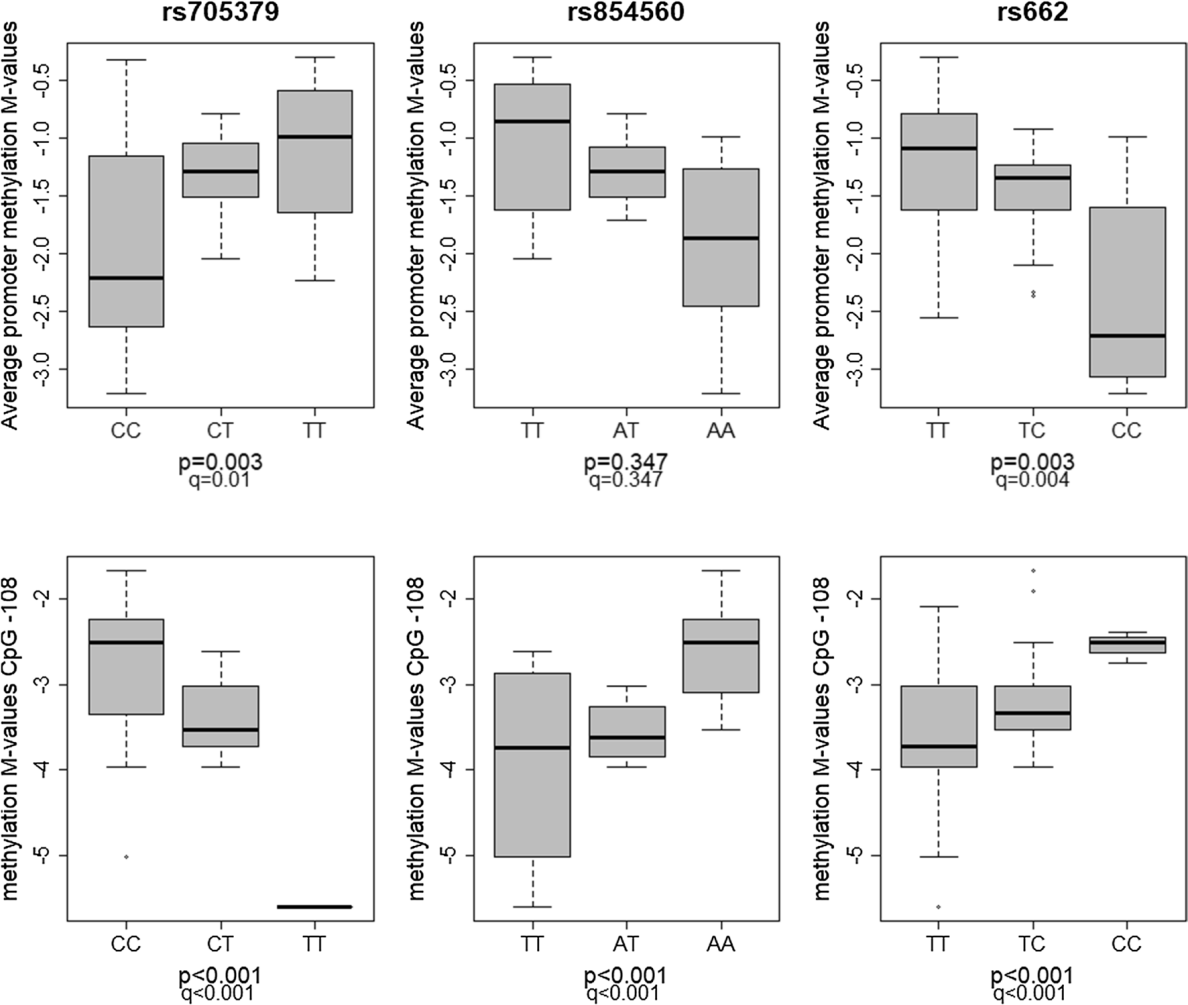
*Cis*-methylation quantitative trait loci relationship. The different boxplots indicate the relationship of the three common *PON1* polymorphism associated CpG sites rs705379:C > T, rs854560:A > T, rs662:T > C in a population of patients with a wide range of (hepato)metabolic derangements. A total of 45 liver biopsies were analysed for which genotype distribution over the three PON1 variants is as follows: 27% CC, 58% CT and 15% TT for rs705379:C > T; 22% TT, 42% AT and 36% AA for rs854560:A > T; and 47% TT, 42% CT and 11% CC for rs662:T > C. Methylation is represented as *M*-values; positive *M*-values mean that more molecules are methylated than unmethylated (> 50% methylation) while negative *M*-values mean the opposite (< 50% methylation). Significance is determined by linear regression after which Benjamini–Hochberg is used to limit the false discoveries to 5% (*q* < 0.05)

Besides evaluating the association of epigenetics with PON1 expression and activity, we also screened for methylation quantitative trait loci (meQTLs). Statistics revealed that the screened polymorphisms can be recognized as *cis*-meQTLs; and significant methylation differences were found for all SNPs except for rs854560:A > T on average promoter methylation (Fig. 3; Additional file 1: Table S5 and Additional file 2: S6).

#### Determinants of PON1 variability outlined

The combined effects of genetics and epigenetics as well as the interrelation between expression and activity were examined in the different HEPADIP subcohorts (Additional file 1: Fig. S1; statistical analysis models glm(activity ~ SNPs*methylation*expression), glm(activity ~ expression), glm(expression ~ SNPs*methylation), were simplified and significance determined).

Multivariable regression analysis showed a combined independent effect of genetics and average promoter methylation on both activities. Polymorphic variant rs854560:T (NP_000437.3:p.55Met) was associated with high average promoter methylation and consequently low activity levels. The combination of SNP rs854560 and average promoter methylation together explained 49.7% and 48.6% of variability for lactone- and arylester-hydrolysing activity, respectively. For SNP rs662:T > C, the highest methylation *M*-values and lowest activity was observed in T-allele carriers (NP_000437.3:p.Gln192), together describing 75% of arylesterase status. The determinants for lactonase status were more complex, with sex also having a significant independent impact on enzymatic activity (37.5% variability). The sex-specific effect translates into low lactonase levels and high average methylation in T-allele male carriers. Furthermore, no combined (epi)genetic effect was for the different polymorphisms with *-108* methylation and for rs705379:C > T with average promoter methylation. These findings generally confirmed previous pairwise association testing results (Figs. 1, 2 and 3).

**Fig. 4:**
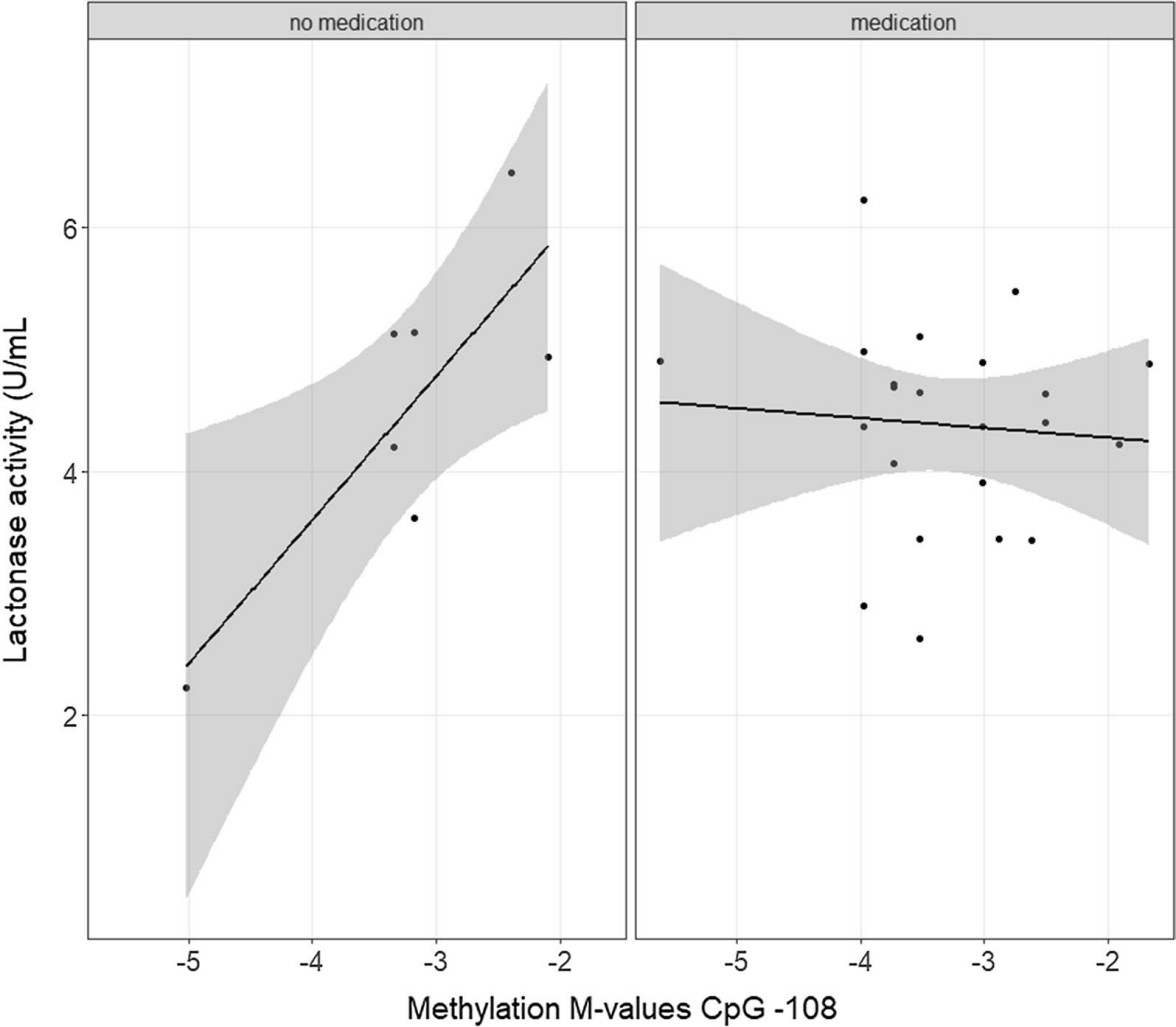
Medication-dependent relationship between *-108* methylation and lactone-hydrolysing activity. The graph represents the combined association of *-108* methylation and medication with lactonase activity in a population of patients with a wide range of (hepato)metabolic derangements. Patients not taking any medication show increased activity levels in case of high methylation. This effect disappears in patients that took medication at the start of the study protocol. Methylation is represented as *M*-values; positive *M*-values mean that more molecules are methylated than unmethylated (> 50% methylation) while negative *M*-values mean the opposite (< 50% methylation). Activity levels towards lactone substrate are expressed as units per millilitre of serum, in which 1 unit equals 1 mmol of 5-thiobutyl butyrolactone hydrolysed/min. Significance was present if *p* < 0.05

In addition, the influence of confounding factors (sex, age, medication, alcohol and smoking habits were added to previous statistical analysis models glm(activity ~ SNPs*methylation*expression), glm(activity ~ expression), glm(expression ~ SNPs*methylation) and simplified to determine significance) was tested and designated a medication-dependent effect of *-108* methylation on lactonase activity (*p* = 0.008, EE = − 1.26 change in enzymatic activity in relation to increased methylation in case medication was taken; STDE = 0.436; *N* = 28). Patients not on medication at the start of the HEPADIP project showed higher activity levels in case of higher methylation (previously mentioned and shown in Figs. 2 and 4). However, this effect (explaining 28.9% of the enzymatic variability) was no longer apparent if a patient took any of the medicine types examined in our population (see Sect. 5.7 for all medical treatment variables considered).

The correlation between PON1 activity and transcript expression was tested. Although we observed a positive trend between *PON1* expression level and lactonase activity (*p* = 0.09; EE = 1.23 change in lactonase activity in relation to increased expression; STDE = 0.7059), no direct relationship between relative expression and arylesterase activity was determined (*p* = 0.124; EE = 1.32; STDE = 0.8173). Moreover, no significant correlation was identified for the combined effect of *PON1* genetics and epigenetics on expression (Additional file 2: Table S6). The same applied for both enzymatic activities concerning expression and *PON1* genetic factors. Significance was not reached, possibly as a result of low sample size (*N* ≤ 22; Additional file 1: Fig. S1).

### PON1 status and obesity-associated liver abnormalities

#### Regulatory polymorphism rs705379:C as a risk variant for NAFLD severity

**Fig. 5:**
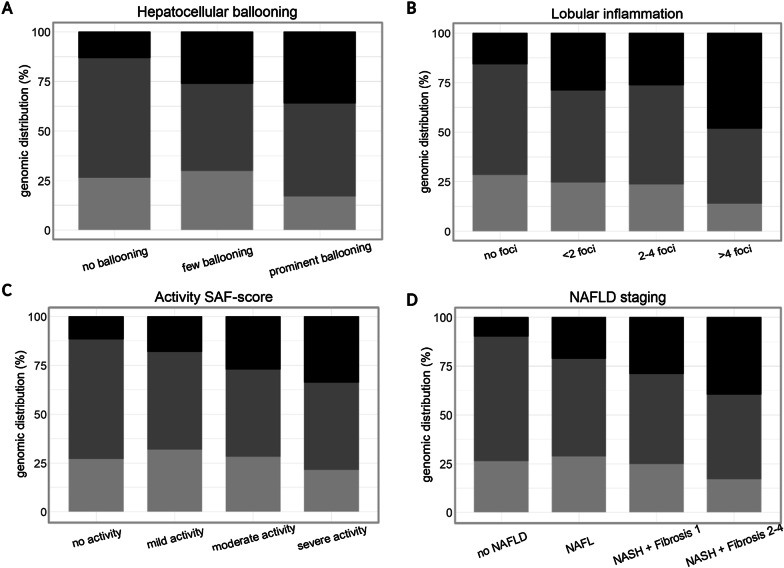
Genomic distribution of regulatory polymorphism rs705379:C > T in relation to histological liver features. The association of liver histology parameters hepatocellular ballooning (**a**), lobular inflammation (**b**), adjusted SAF activity (**c**), and NAFLD staging (**d**) with common SNP rs705379:C > T is shown in a population of patients with a wide range of (hepato)metabolic derangements. The different categories of each histological liver feature are represented on the *x*-axis. The distribution of each genotype in the HEPADIP population is exposed as a percentage on the *y*-axis. Light grey to black shading represents respectively genotypes TT, CT, CC

Pairwise association testing was performed to explore PON1 status in relation to the hepatometabolic phenotype (all variables listed in Table 1 were selected as response variable while the different PON1 levels, including SNPs, methylation, expression and activity, were selected as explanatory variable). A direct correlation between promoter SNP rs705379:C > T and different histological findings was shown by statistical analyses (Fig. 5). Patients carrying the C-allele showed more prominent hepatocellular ballooning (*p* < 0.001; *q* = 0.005), higher lobular inflammation (*p* = 0.02; *q* = 0.06), and more severe activity (*p* = 0.003; *q* = 0.026). Accordingly, homozygous C-allele carriers were diagnosed with a more progressive NAFLD phenotype (*p* = 0.01; *q* = 0.04). Their odds of being diagnosed with isolated steatosis (i.e. NAFL) over no NAFLD was 5.4 times higher (*p* = 0.01, 95%CI 4.13–6.74) and even increased to an odds ratio of 15.9 and 10 for NAFLD categories “NASH + fibrosis grade 1” (*p* < 0.001, 95%CI 14.76–17.14) and “NASH + fibrosis grade 2–4” (*p* < 0.001, 95%CI 8.70–11.23), respectively. This was counteracted in case the patient was taking medication (see Sect. 5.7 for all medical treatment variables considered). Subsequently, a 0.27 fold decrease of being diagnosed with NAFLD category “NASH + fibrosis grade 2–4” (*p* = 0.01, 95%CI 0.04–2.23) was observed in homozygous C-allele carriers.

#### Vertical data integration Of PON1: *omics* levels exposes a link with obesity-related liver disease

Integrative analysis of the different PON1 -*omics* data layers (Additional file 1: Fig. S1; all variables listed in Table 1 were selected as response variable while the different PON1 levels, in combination with confounding factors such as age and sex, were selected as explanatory variable) demonstrated a significant positive relationship between PON1 activity (both for arylesterase and lactonase) and lipid parameters HDL and total cholesterol (*q* < 0.01, *q* < 0.01, EE_chol-aryl_ = 5.13, EE_chol-lac_ = 6.16, EE_HDL-aryl_ = 2.41, EE_HDL-lac_ = 3.57 increase in relation to higher PON1 levels; Additional file 1: Fig. S7). The association with HDL was further independently influenced by sex; males tended to have lower HDL levels compared to females (*p* < 0.001). Together, enzymatic activity and sex explained 16.8–19.1% of HDL variability. Furthermore, determination of the methylation level at the *PON1* promoter region showed a significant positive relationship at CpG site *-108* with gamma-glutamyltransferase (GGT; *p* = 0.003, EE = 0.3507 change in GGT in relation to increased *-108* methylation; STDE = 0.1088; *q* = 0.049).

**Fig. 6:**
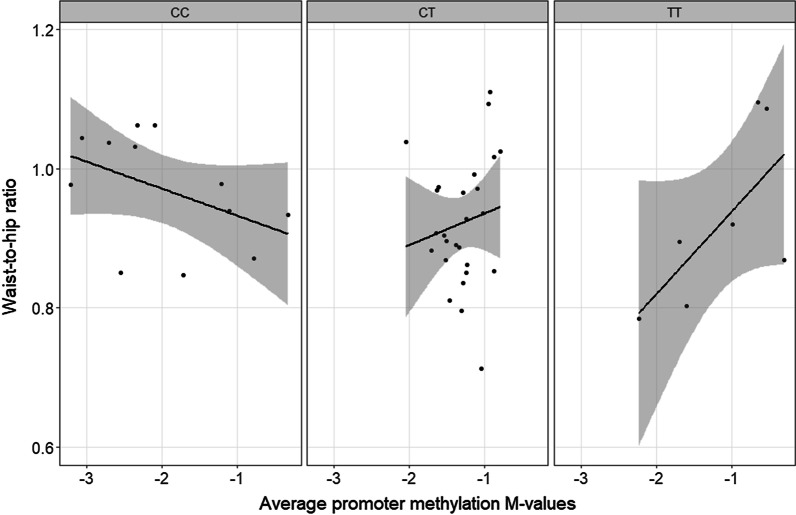
Correlation between PON1 variability and waist-to-hip ratio. The graph represents the combined (epi)genetic relationship between rs705379:C > T and average promoter methylation on waist-to-hip ratio in a population of patients with a wide range of (hepato)metabolic derangements. Average promoter methylation is displayed as methylation *M*-values on the *x*-axis and waist-to-hip ratios on the *y*-axis. The three different screens in the graph illustrate the genotypes of rs705379:C > T. A total of 45 liver biopsies were analysed for which genotype distribution over PON1 rs705379:C > T is 27% CC, 58% CT and 15% TT. Methylation is represented as *M*-values; positive *M*-values mean that more molecules are methylated than unmethylated (> 50% methylation) while negative *M*-values mean the opposite (< 50% methylation)

Multivariable statistics combining *PON1* polymorphisms with methylation data illustrated an association with Waist-to-Hip Ratio (WHR). An interaction between rs705379:C > T and average promoter methylation was exposed by multivariable statistics and indicates that patients homozygous for the C-allele show high WHR when average promoter methylation is low. In contrast, heterozygous patients and patients homozygous for the T-allele have high WHR if hypermethylation was present (Fig. 6; *p* = 0.006). A sex-specific effect (33%; *p* < 0.001; EE = 0.13 WHR increase in female patients; STDE = 0.027505) was also found, which together with the combined (epi)genetic relationship (13.9%; EE = 0.006 WHR increase in T-allele female carriers with increased average promoter methylation; STDE = 0.002), described 46.9% of the variability in WHR.

No direct or combined associations were found between PON1 variability and BMI.

## Discussion

A targeted multi-*omics* approach was applied to examine the role of PON1 in relation to obesity-associated fatty liver disease. Different levels of variability including *PON1* genetic polymorphisms, DNA methylation and confounding factors such as sex, medication, etc. were considered to understand the mechanisms that regulate its expression level and enzymatic activity. Identification of signatures that define PON1 status could be valuable to clarify their influence on the pathophysiology of hepatometabolic disease.

Pairwise association testing revealed a correlation between the regulatory polymorphism rs705379:C > T (NM_000446.6;c.-108C > T) and different histological liver features (Fig. 5). More specifically, our results indicate that patients carrying the C-allele are at a higher risk of being diagnosed with NAFLD and the more severe subtype NASH. A molecular basis for the this relationship can be found in the location of this variant on the *PON1* locus. Risk variant rs705379:C is positioned in a site within the promoter region of *PON1* known to bind transcription factor Specific protein 1 (Sp1) in an interactive manner with sterol regulatory element-binding protein-2 (SREBP-2). This latter protein plays a central role in lipid metabolism by the regulation of cellular cholesterol biosynthesis, uptake and excretion [34, 35]. Overexpression of *SREBP-2* has also been associated with free cholesterol accumulation in the liver and consequently NAFLD pathogenesis [36]. Particularly, liver disease severity seems to be strongly related to dysregulation of *SREBP-2* as animal and clinical studies have demonstrated that alterations in transcriptional regulators of cholesterol homeostasis mediate the progression of steatosis to steatohepatitis [37–39].

**Fig. 7:**
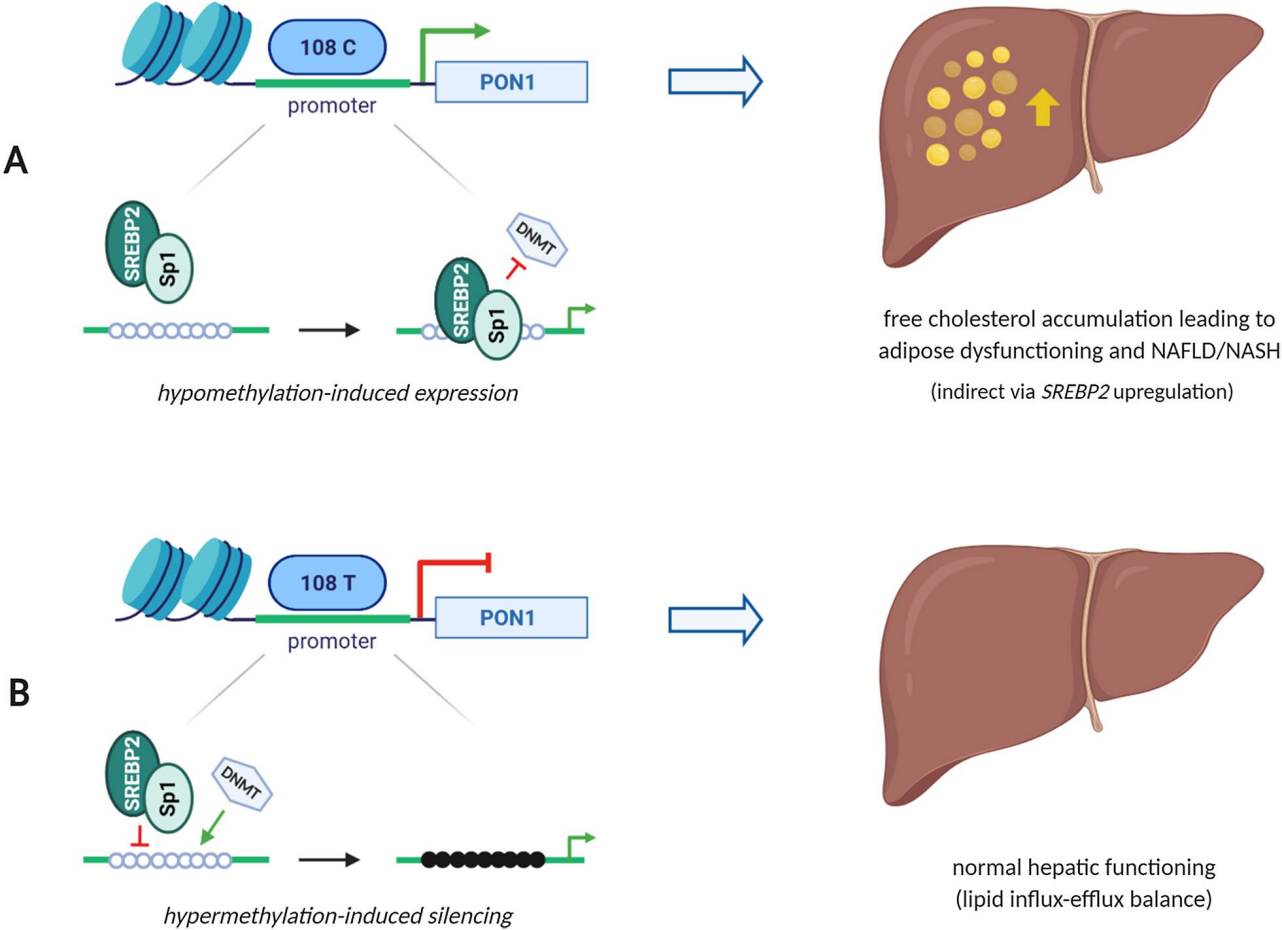
Molecular mechanism proposed for PON1 in NAFLD pathophysiology. Schematic representation of the suggested gene-regulatory effect of rs705379:C > T on NAFLD pathogenesis. The PON1 polymorphism is located in the promoter region of PON1 and identified to bind transcription factor Specific protein 1 (Sp1) in an interactive manner with sterol regulatory element-binding protein-2 (SREBP-2). Sp1 is known to initiate *PON1* transcription in a methylation-dependent manner while SREBP-2 is implicated in cholesterol homeostasis. Two different scenarios are possible depending on which polymorphic variant is present. **a** SNP rs705379:C results in hypomethylation-induced expression and activity of PON1. Simultaneously, *SREBP-2* upregulation is observed resulting in hepatic cholesterol accumulation and increased susceptibility for NAFLD/NASH development. **b** SNP rs705379:T alters transcription factor binding affinity thereby resulting in hypermethylation-induced silencing, normal SREBP-2 levels and correct functioning of the liver. Figure was created with BioRender.com

Thorough examination of the different PON1 -*omics* layers enables us to postulate about the involvement of complex (epi)genetic mechanisms and gene expression modulation as an explanation for the observed *PON1* association with NAFLD severity. Polymorphism rs705379:C > T can be observed as a CpG-SNP, which is known to introduce or remove a CpG site at position *-108*. The outcome is a drastic change in the promoter regulatory status through adaptation of the substrate. This is reflected by hypermethylation-induced expression and activity perceived in C-allele carriers versus T-allele carriers (Figs. 1 and 3). Additionally, analysis has shown that CpG-SNPs not only interfere with the DNA methylation status of their own CpG site but that they can also alter methylation patterns of CpG sites in close proximity [15]. Such a relationship was found for rs705379:C > T with average *PON1* promoter methylation in our patient cohort (Fig. 3). The significant association of average promoter hypermethylation both with reduced PON1 expression (Additional file 1: Fig. S6) and both enzymatic activities (Fig. 1) suggests that alterations in gene expression and catalytic efficiency could be the intermediate step between (epi)genetics and the disease phenotype. Research designated that methylation-disrupting SNPs can change expression and/or activity by different mechanisms (e.g. alternative splicing, transcription factor binding, protein folding). A plausible explanation for our results is the regulation of gene expression through the perturbation of transcription factor binding site (TFBS) affinity. TFBS analysis indicated that position -*108* occurs in the recognition sequence of transcription factor Sp1 [40, 41]. Confirmation of this finding was provided by a study by Deakin et al. [42], which demonstrated a complex formation between Sp1 and the -*108* polymorphic site. Moreover, it was demonstrated that overexpression of *Sp1* enhances *PON1* transcription and that this is mediated by methylation [30, 43–45]. Sp1 has thus been shown to bind unmethylated regulatory regions thereby preventing DNA methyltransferases from accessing the promoter and activating transcription [46]. Perturbation of TFBS affinity is found when a cytosine is replaced by a thymine at position -*108*. In this case, the recognition sequence for Sp1 is disrupted, hypermethylation is present and transcriptional repression is observed. In accordance with Huen et al. [30], we believe that this gene-specific transcriptional silencing might explain the genotype differences of rs705379:C > T (NM_000446.6;c.-108C > T) with average promoter methylation and PON1 status. Accordingly, the C > T nucleotide substitution will disrupt the interaction between proteins Sp1, SREBP-2 and the *PON1* promoter (Fig. 7). This was reflected in our cohort by a decrease in enzymatic activity (arylesterase and lactonase) and disease risk, and suggests that upregulation of *SREBP-2* and high PON1 activity both promote NAFLD pathogenesis.

Our hypothesis is supported by the association observed between -*108* hypermethylation and increased plasma GGT levels. GGT is one of the hepatic enzymes traditionally used in first-line indicative tests to predict NAFLD development [47]. Alterations in GGT levels have been associated with markers of chronic inflammation (e.g. fibrinogen) and oxidative stress (e.g. lipid peroxides), both hallmarks of NAFLD progression [48, 49]. Our previously identified relationship between -*108* hypermethylation and increased arylesterase activity (Fig. 2) thus indicates that the presence of high PON1 levels in the blood is associated with increased GGT and high-risk disease prevalence. Likewise, the observed medication-dependent effect on NAFLD staging (0.27 fold decrease in being diagnosed with severe NASH) was associated with *-108* methylation and consequently lactonase activity (Fig. 4). Patients not taking any medication showed increased activity levels in case of *-108* hypermethylation. Medication-taking behaviour abolished this relationship and indicates the importance of epigenetics as an intermediate between environmental factors and PON1 status. Various pharmaceuticals have been reported to modify methylation patterns in gene promoter regions, thus affecting gene expression and protein levels. Our results thus suggest that the previously mentioned hypermethylation-induced transcriptional activation will be altered as a result of drug exposure, and thereby reducing the odds of a more progressive NAFLD diagnosis. The finding that a higher WHR is measured in C-allele carriers of SNP rs705379 with decreasing methylation levels reinforces the (indirect) link between high PON1 activity and NAFLD pathogenesis (Fig. 6). Our formerly observed inverse correlation between average *PON1* promoter methylation and both enzymatic activities (Fig. 2) shows that this increase in WHR is likewise associated with elevated PON1 levels. The knowledge that WHR is an important sex-specific metric in the evaluation of weight-related disease [50], proposes the rs705379:C (NM_000446.6;c.-108C) variant and consequently high PON1 activity as a determinant for hepatic manifestations in patients affected by obesity rather than a risk factor for the onset of metabolic disease. This is confirmed by the non-significant correlation between BMI and the different PON1 -*omics* data layers in our study population (Additional file 2: Table S6) and is consistent with the current insights into NASH pathophysiology, which indicate the importance of elevated visceral-to-subcutaneous adipose tissue ratios (cfr. WHR) over a general increase in body weight (cfr. BMI) as an important mechanism of NAFLD initiation and NASH progression [51].

The strength of our experimental design is the extensive examination of multiple PON1 -*omics* data layers in a large hepatometabolic cohort. It facilitated us to perform a targeted multi-*omics* approach that enabled us to investigate the relationship between PON1 status and obesity-associated liver pathology as well as to explore the potential involvement of *PON1* (epi)genetics in the regulation of its gene expression and catalytic efficiency. PON1 is known to be secreted in the blood circulation where it associates with HDL. This was supported by the observed positive association of PON1 status with cholesterol and HDL. The general assumption is that the presence of both HDL and apolipoprotein A1 (apoA1) are necessary to bind PON1 with high affinity. The binding of HDL particles carrying apoA1 occurs with the *N*-terminal domain of PON1, thereby stabilizing the enzyme and stimulating its activity [52, 53]. Physiological distribution and PON1 status are thus dependent on the direct binding to phospholipids in association with apoA1. Moreover, we were able to determine the importance of *PON1* genetic and epigenetic variation in relation to its status. A genotype-dependent difference was observed for promoter methylation (i.e. meQTL), which further translates into changes in both enzymatic activities (Figs. 1 and 3). This finding suggests that methylation acts as a mediator between *PON1* genetics and PON1 activity. Integration of genetic and epigenetic data confirms this assumption and reveals a combined independent effect for rs854560:A > T and rs662:T > C with average promoter methylation on PON1 status (Fig. 2). High average promoter methylation and low activity levels were detected for T-allele carriers of coding polymorphisms rs854560 (NP_000437.3:p.55Met) and rs662 (NP_000437.3:p.Gln192), independent of the hydrolysed substrate. The relationship that was identified can be ascribed to conformational changes in the three-dimensional structure of the enzyme. The rs854560:A > T (NP_000437.3:p.Leu55Met) polymorphism has been associated with alterations in protein stability and consequently PON1 concentration, while rs662:T > C (NP_000437.3:p.Gln192Arg) has been shown to be part of PON1’s catalytic site thereby regulating its enzymatic activity in a substrate-dependent manner [54–56].

A limitation in our study was the restricted availability of RNA samples for gene expression analysis. As we only had 22 biological replicates available, the sample size was too small for a representative *PON1* expression distribution over the different genotypes. Consequently, we could not validate the assumption of gene expression modulation as the intermediate mechanism between regulatory variant rs705379:C > T, alterations in TFBS affinity and NAFLD severity. The same limitation applies for pairwise testing of *PON1* expression with enzymatic activity (*q* > 0.05). Despite the fact that we found increased promoter methylation to be negatively associated with expression (*q* = 0.02; Additional file 1: Fig. S6A) and that similar relationships between (1) *PON1* genetics and expression (Additional file 1: Fig. S2) versus *PON1* genetics and activity (Fig. 1) and (2) epigenetics and expression (Additional file 1: Fig. S6) versus epigenetics and activity (Fig. 2) were observed, future experiments in a larger sample set will be necessary to demonstrate its significance and to validate the intuitive hypothesis that gene expression is a major determinant of PON1 status.

Although our study consists of an extensively monitored large obesity cohort with(out) the presence of hepatic manifestations, we only had restricted information on other variables known to influence PON1 status. PON1 is recognized as a multifunctional protein that is modulated by many different exogenous factors (e.g. chemicals, lifestyle, diet). Likewise, its activity can vary depending on different physiological conditions or pathological states [57]. It is therefore difficult to perform studies on PON1 that include all potential confounding factors and caution should be taken for result interpretation, as our study did not include information on the patients’ cardiovascular and neurological states (two disease states known to be correlated with PON1 status) [13, 14].

Another drawback is the cross-sectional nature of our study, as it does not allow us to determine whether the relationship we found between high PON1 activity and hepatic manifestations was a cause or a consequence of obesity and/or NAFLD. Two possible explanations exist that can describe our results being (1) high PON1 activity is a driving force in the development of obesity-associated NAFLD or (2) high PON1 activity is a consequence of obesity and its comorbidity NAFLD. This latter explanation is most consistent with our results and best reflects the general idea that an increase in PON1 exhibits a protective role against the susceptibility for obesity and associated fatty liver disease [28–31]. The observed increase in PON1 activity levels would in this manner reflect a defence mechanism to prevent further hepatometabolic manifestations that lead to the advancement of obesity to NAFLD/NASH. In this manner, our results can be considered as a pilot study indicating the importance of integrating multiple levels of data variation to unravel complex disease mechanisms.

## Conclusions

Vertical data-integration of PON1 genetic polymorphisms, epigenetic DNA methylation variation, gene expression profile and enzyme activity revealed a possible molecular mechanism through which the three most frequently studied polymorphisms may influence PON1 status (high activity levels for rs705379:C, rs854560:A and rs662:C). Changes in the epigenetic profile in relation to the genotype propose that methylation acts as a mediator between *PON1* genetics and enzymatic activity. The most pronounced effect observed in the present study is a reduction in PON1 levels resulting from average promoter DNA hypermethylation. However, local hypermethylation-induced PON1 activity was also found. Polymorphisms in the coding region of *PON1* are attributed to conformational changes with a direct influence on its catalytic efficiency. The relationship between the regulatory CpG-SNP rs705379:C > T and PON1 status is more challenging with a potential involvement of gene expression modulation via Sp1 (Fig. 7). Our findings suggest that increased binding of *Sp1* enhances *PON1* transcription mediated by hypomethylation. Introduction or removal of a CpG site at position -*108* consequently leads to overall transcriptional repression and lower PON1 levels. Furthermore, it is known that Sp1 can bind the promoter region of *PON1* in an interactive manner with SREBP-2. Overexpression of *SREBP-2* has been associated with hepatic cholesterol accumulation and NAFLD severity. This was reflected in our cohort by the significant correlation between rs705379 C-allele carriers and the higher odds of being diagnosed with a more progressive NAFLD phenotype. We therefore believe that PON1 activity is an important contributor to the pathophysiology of NAFLD/NASH. This assumption was further reinforced by the found association that was found of PON1 status with increased GGT levels and WHR.

Our study results illustrate the relevance of performing integrative multi-*omics* approaches to gain insights into the crosstalk of different levels of variation in gene function and disease phenotypes. Future longitudinal studies on large obesity cohorts will be necessary to shed light on the causality of the association observed between PON1 status and hepatometabolic disease.

## Methods

Patients visiting the obesity clinic of the Antwerp University Hospital (a tertiary referral facility) for a problem of overweight (BMI ≥ 25–29.9 kg/m^2^) or obesity (BMI ≥ 30 kg/m^2^), presenting at their own initiative or referred by their treating physician, were consecutively recruited. Every patient underwent a standard metabolic work-up combined with a liver-specific program [58, 59], both approved by the Ethics Committee of the Antwerp University Hospital (reference 6/25/125, Belgian registration number B30020071389) and requiring written informed consent of the patient.

### Metabolic work-up

Anthropometric measurements were carried out in the morning, with patients in fasting conditions and undressed. Height was measured to the nearest 0.5 cm and body weight was measured with a digital scale to the nearest 0.2 kg. BMI was calculated as weight (in kg) divided by height (in m) squared. Waist circumference was measured at the mid-level between the lower rib margin and the iliac crest. Hip circumference was measured at the level of the trochanter major. The WHR was calculated by dividing waist circumference by hip circumference. Body composition was determined by bio-impedance analysis as described by Lukaski et al. [60] and body fat percentage was calculated by the formula of Deurenberg et al. [61]. The cross-sectional areas of total abdominal adipose tissue, visceral abdominal tissue and subcutaneous abdominal adipose tissue were measured by a CT-scan at L4-L5 level [62]. Systolic and diastolic blood pressure were determined on the right arm of the patient, after at least 5 min rest, using a mercury sphygmomanometer. Fasting blood samples were taken from an antecubital vein and collected into BD Vacutainer EDTA tubes (Becton Dickinson Medical Devices Co Ltd, Benelux). Serum samples were acquired by blood collection into BD Vacutainer serum separator tubes (Becton Dickinson Medical Devices Co Ltd, Benelux). Lipid profiles including total cholesterol, HDL cholesterol, LDL cholesterol and triglycerides were measured. Levels of liver enzymes [aspartate aminotransferase (AST), alanine aminotransferase (ALT), alkaline phosphatase (ALP), gamma-glutamyltransferase (GGT)] and creatinine kinase were measured. The insulin resistance estimation was assessed using the homeostasis model and was calculated as [insulin (mU/L) × glucose (mmol/L)]/22.5 [63].

### Hepatological work-up

Additional blood analyses were performed to exclude classical aetiologies of liver disease (e.g. viral hepatitis and autoimmune disease). The metabolic liver reserve was determined by an aminopyrine breath test and a Doppler ultrasound of the abdomen. The ultrasound appearance of the liver parenchyma was scored by making the sum of the echogenicity of the liver parenchyma compared to the renal parenchyma (0: no hyperechogenicity; 1: mild-to-moderate hyperechogenicity; 2: moderate-to-severe hyperechogenicity) and posterior beam attenuation (0: absent; 1: present) resulting in an ultrasound steatosis score (USS) ranging 0–3. Patients were excluded from the study in case of (1) diagnosis of another liver disease than NAFLD, (2) significant alcohol consumption (> 20 g/day) and/or (3) history of bariatric surgery. Longstanding diabetes is a known risk factor for fibrosis and some therapies and interventions for diabetes are known to have an impact on NAFLD pathology. Accordingly, patients previously diagnosed with diabetes were not included as diabetes and its associated treatments were considered major confounding factors for NAFLD pathogenesis. If conversely diabetes was de novo diagnosed, patients were not excluded as de novo diagnosis is not considered as a confounder for the expected results of the liver biopsy. Glucose tolerance status was defined based on the criteria of the American Diabetes Association [64].

#### Liver biopsy

Patients with suspicion of NAFLD, defined by abnormal liver tests (AST and/or ALT and/or GGT and/or ALP) and/or liver ultrasound abnormality (steatotic liver defined by USS ≥ 1), were offered a liver biopsy for accurate diagnosis (gold standard). Subsequently, they entered a weight-management program. Some patients underwent bariatric surgery, for whom a liver biopsy was proposed regardless of the criteria. Upon informed consent, a liver biopsy was performed percutaneously (16G Menghini), transjugularly (16G transjugular liver biopsy needle) or perioperative (16G Trucut needle). Haematoxylin–eosin stain, Sirius red stain, reticulin stain and Perl’s iron stain were routinely performed on all biopsies.

Different histological features were blindly assessed by two experienced pathologists. NAFLD subtype and severity were defined by use of the NASH Clinical Research Network (NASH-CRN) scoring system [65–67]. They proposed the NAFLD Activity Score (NAS), which evaluates NAFLD lesions by summing component scores for steatosis, lobular inflammation, and hepatocellular ballooning (score ranges from 0 to 8). Despite the fact that this scoring system is widely used by both clinical and research communities, the FLIP-algorithm was suggested in 2012 to evaluate liver biopsies of patients with morbid obesity [68]. In this scoring system, steatosis (S) is scored separately from the activity (A, sum of lobular inflammation and hepatocellular ballooning), resulting in the SAF score (F = fibrosis). As differences are present between both scores in the definition for the grading of hepatocellular ballooning and lobular inflammation, we introduced the adjusted SAF activity (aSAF-A) in our dataset. This variable is an equivalent of the activity parameter used in the FLIP-algorithm and calculated as the sum of lobular inflammation and hepatocellular ballooning scored by the NASH-CRN system. Lastly, we introduced the NAFLD staging parameter used to subclassify the patient population into (1)“no NAFLD”, (2) “NAFL”, (3) “NASH + fibrosis grade 1” and (4) “NASH + fibrosis grade 2–4”.

The complete study protocol was performed according to the Declaration of Helsinki and approved by the Ethics Committee of the Antwerp University Hospital. All authors had access to the study data and had reviewed and approved the final manuscript. An overview of patient samples is presented in Additional file 1: Fig. S1. Anthropometric and biochemical values analysed in the population are summarized in Table 1.

### SNP genotyping

Polymorphisms located on the paraoxonase-1 (*PON1)* locus are indicated as genetic determinants of PON1 variability [30]. Although more than 400 SNPs have been described on chromosomal region 7q21.3, most of the variability can be explained by polymorphisms in the 5’ regulatory region (NM_000446.6;c.-108C > T) and the coding region (NP_000437.3:p.Leu55Met and NP_000437.3:p.Gln192Arg) [69, 70]. The three common polymorphism rs705379:C > T (NM_000446.6;c.-108C > T), rs854560:A > T (NP_000437.3:p.Leu55Met) and rs662:T > C (NP_000437.3:p.Gln192Arg) were genotyped in the complete HEPADIP patient cohort (*N* = 790; Additional file 1: Fig. S1). Predesigned TaqMan SNP Genotyping Human Assays were used to study the selected SNPs (Applied Biosystems Inc., CA, USA). Genomic DNA was extracted from whole blood samples. Allelic discrimination was performed according to the manufacturer's protocol with the use of the Lightcycler 480 Real-Time PCR System (Roche, Penzberg, Germany). Quality control was performed by Sanger sequencing three independent samples for each genotype of the analysed PON1 polymorphisms.

### Pyrosequencing

Integrative analysis of genetic and epigenetic data holds promise to better understand the pathogenesis of complex diseases such as obesity and NAFLD [15, 71]. Genotype-specific alterations in DNA methylation may be related to adverse metabolic risk via negative *PON1* modulation [27, 72]. For this reason, we assessed DNA methylation status at the *PON1* promoter region in a subcohort of the patient population (*N* = 45; Additional file 1: Fig. S1). To select a *cis*-MeQTL *PON1* region of interest for DNA methylation quantification by pyrosequencing, we prioritized the CpG island promoter region, localized in a transcriptional permissive “weak promoter enhancer” chromatin state [73], previously associated with control of gene expression [74].

Briefly, genomic DNA was extracted from the liver using the DNeasy Blood & Tissue Kit (Qiagen, Hilden, Germany) and subjected to bisulphite conversion using the EpiTect® Fast Bisulfite Conversion kit (Qiagen, Hilden, Germany). A pyrosequencing approach was applied to measure DNA methylation levels. In summary, amplification of the bisulfite treated DNA fragments was performed using the PyroMark PCR kit (Qiagen, Hilden, Germany). Immobilization, capturing and denaturation of biotin-labelled amplicons was performed using the PyroMark vacuum Q24 workstation (Qiagen, Hilden, Germany). Single stranded PCR products were annealed with the sequencing primer and pyrosequenced on a PyroMark Q24 instrument (Qiagen, Hilden, Germany). DNA methylation of each CpG site was quantitatively assessed using the PyroMark Q24 Advanced software (Qiagen, Hilden, Germany). The Pyro Q-CpG software delivers a quality control report for each sequencing run and only assays which “pass” software quality control requirements were considered. Blank dispensations did not exhibit any intensity signal being significantly above background signals. Software Assay “pass” quality settings confirmed that gDNA was completely bisulfite converted by bisulfite conversion controls at a specific locus (C > T in non CpG context) and unconverted C signal was close to background intensity and did not cross minimal signal threshold. The signal intensity of the expected peaks was at least 30–50 RLU, to be distinguished from background signals [75]. Methylation levels were defined by methylation values (*M*-values) calculated as the log2 ratio of the intensity signal obtained from the methylated allele versus the unmethylated allele [76]. Assays and biotinylated-reverse, forward and sequencing primers were designed with PyroMark Assay Design 2.0 software (Qiagen, Hilden, Germany).

### Gene expression

*PON1* gene expression was examined in 22 patients of the HEPADIP cohort for which high quality RNA was available (Additional file 1: Fig. S1). RNA was isolated from human liver biopsy by acid guanidinium thiocyanate-phenol–chloroform extraction [77]. Single-stranded cDNA was synthesized using the High Capacity cDNA Reverse Transcription kit (Applied Biosystems Inc., Foster City, CA, USA). Quantitative real-time PCR was performed with Brilliant II SYBR Green QPCR Master Mix on a Stratagene Mx3005P system (Agilent Technologies, SC, USA). Relative changes in *PON1* expression (NM_000446) were normalized to the RNA levels of three housekeeping genes according to the 2^ −ΔΔCt^ method[78]. cDNA primers were designed with the primer-BLAST tool of NCBI. Qualitative gene expression data was guaranteed by the inclusion of negative and no template controls, biological and technical triplicates and reference genes as internal control for normalization.

### Activity measurements

PON1 is an ambiguous enzyme capable of hydrolysing a wide variety of substrates including thiolactones, arylesters, organophosphorus pesticides, nerve gases, estrogen esters and lipoproteins [25]. Accordingly, identifying its physiological relevance and native substrate have long been the focus of many studies. Structure–reactivity experiments suggest a native enzymatic activity of PON1 towards lactones with fatty acid oxidation as its major biological function [23, 79, 80]. Paraoxonase and arylesterase are in contrast promiscuous activities able to hydrolyse numerous man-made chemicals [81]. For our study, we determined substrate-specific lactonase and arylesterase activities since the combination most accurately reflects the concentration and activity of PON1 [49, 54]. Paraoxonase activity was not considered as extreme differences (> 40 fold) are apparent for paraoxon hydrolysis in relation to genetic polymorphisms [25].

Serum lactonase activity was evaluated by measuring the hydrolysis of 5-thiobutyl butyrolactone (TBBL). This method involves the use of a chromogenic lactone structurally resembling the proposed natural lipolactone substrate. Serum samples were prepared in sample buffer containing 50 mmol/L Tris and 1 mmol/L CaCl_2_ (pH = 8.0) in a 20-fold dilution. Each well of a 96-well plate was loaded with 1 μL 100 mmol/L 5,5′-dithio-bis-2-nitrobenzoic acid in dimethyl sulfoxide, 45 μL of 4% acetonitrile solution in sample buffer, 5 μL of diluted serum, and 50 μL of sample buffer. The kinetic reaction was initiated by adding 100 μL of substrate buffer, consisting of 0.4 mmol/L TBBL solution in sample buffer, to each well of the 96-well plate. The reaction was monitored at 412 nm in an automated microplate reader at 37 °C (PowerWave XS, Bio-Tek Instruments Inc., Winooski, VT, USA). Spontaneous TBBL hydrolysis, defined as the hydrolysis rate in the absence of serum, was subtracted from all measurements. Lactonase activity was expressed as units per millilitre of serum, in which 1 unit equals 1 mmol of TBBL hydrolysed/min. The molar extinction coefficient used to calculate the rate of hydrolysis was 7000 M^−1^ cm^−1^. A path-length correction was applied for the use of microtiter plates.

Serum arylesterase activity was quantified by measuring the hydrolysis of *p*-nitrophenyl acetate to *p*-nitrophenol. The working reagent consisted of 50 mmol/L Tris–HCl (pH = 8.0) with 1 mmol/L CaCl_2_. The substrate was prepared in water as a separate starting reagent (2.5 mmol/L *p*-nitrophenyl acetate). The formation rate of *p*-nitrophenol was evaluated by adding 2 μL undiluted sample to 300 μL of the working reagent, after which 72 μL of starting reagent was added to initiate the kinetic reaction. The change in absorption was monitored at 405 nm at 37 °C in an automated biochemistry analyser (Olympus AU600, Olympus Europe GmbH, Hamburg, Germany). The non-enzymatic hydrolysis of *p*-nitrophenyl acetate, based on the hydrolysis rate in the absence of serum, was subtracted from the total hydrolysis rate. Arylesterase activity was expressed as units per millilitre of serum, in which 1 U equals 1 μmol of phenyl acetate hydrolysed/min. The molar extinction coefficient used to calculate the rate of hydrolysis was 14,000 M^−1^ cm^−1^.

### Statistical analysis

Associations between the clinical hepatometabolic phenotype and *PON1* genetic variants (*N* = 790), epigenetic DNA methylation variation (*N* = 45), gene expression profile (*N* = 22) and enzymatic activity (*N* = 714) were examined in the HEPADIP cohort. Simultaneously, the occurrence of *cis*-meQTLs was assessed by looking whether the methylation levels of the PON1 promoter were influenced by one of the three examined PON1 polymorphisms. Pairwise comparison testing was performed by a linear regression analysis (continuous variable) or chi-squared test (categorical variable) in the different HEPADIP subsets (Additional file 1: Fig. S1). Both the significance level (*p*) and false discovery rate threshold (*q*, Benjamini–Hochberg) were set at 0.05. The number of tests for multiple testing correction was determined within each aim. Due to the limited availability of patient liver biopsy material of the different PON1 genotypes, genome-wide Illumina 450 K methylation array studies could not applied to verify further associations of PON1 SNPs with previously described *trans*-MeQTLs involved in metabolic health [27].

A targeted multi-omics approach was applied by designing (multinomial) logistic regression models. As the HEPADIP cohort is a highly detailed collection of patient samples with > 50 metabolic and hepatic parameters (including histology), data exploration via principal component analysis (PCA) was performed to identify and remove highly correlated variables (i.e. multicollinearity) from the statistical model. A multivariable regression model was used to study the combined impact of PON1 data parameters on the hepatometabolic phenotype. Confounding factors such as age, sex, medication and smoking/alcohol habits were also added in the model to verify whether PON1 variability independently influences the phenotype or not. PCA identified that all different drug treatment parameters considered in the HEPADIP study population (oral contraceptives, vitamins, hormones, diabetes medication, weight loss medication, blood pressure medication, lipid lowering medication) were highly interconnected, resulting in grouping of these different medication types into one combined single eigenvector (i.e. medical treatment variable.) All statistical analyses were carried out in R version 3.6.1.

All measured parameters (Sect. 5.1–5.6) were observed as explanatory and/or potential confounders for obesity and were as such included in statistical analyses. An overview of all tested correlations with their respective significance level (both significant and non-significant) can be found in Additional file 2: Table S6. Correlations that showed significance are described and discussed in detail throughout Sects. 2 and 3 of the main document.

## Supplementary Information

**Additional file 1.** Supplementary tables and figures.

**Additional file 2.** Supplementary statistics table.

## Data Availability

Most data generated or analysed during this study are included in this published article and its Additional files. Additional datasets used and/or analysed during the current study are available from the corresponding author on reasonable request.

## Abbreviations

ALP: Alkaline phosphatase
ALT: Alanine aminotransferase
apoA1: Apolipoprotein A1
AST: Aspartate aminotransferase
BMI: Body mass index
GGT: Gamma-glutamyltransferase
HEPADIP: Hepatic and adipose tissue and functions in the metabolic syndrome
HDL: High-density-lipoproteins
LDL: Low-density-lipoproteins
meQTL: Methylation quantitative trait locus
M-values: Methylation values
NAFLD: Non-alcoholic fatty liver disease
NAS: NAFLD activity score
NASH: Non-alcoholic steatohepatitis
NASH-CRN: Non-alcoholic steatohepatitis clinical research network
PCA: Principal component analysis
PON1: Paraoxonase-1
SNP: Single nucleotide polymorphism
Sp1: Specific protein 1
SREBP-2: Sterol regulatory element-binding protein-2
TBBL: 5-Thiobutyl butyrolactone
TFBS: Transcription factor binding site
USS: Ultrasound steatosis score
WHR: Waist-to-hip ratio
