# Supplementary material for: A targeted multi-omics approach reveals paraoxonase-1 as a determinant of obesity-associated fatty liver disease
Source: Clin Epigenetics. 2021 Aug 13;13:158. doi: 10.1186/s13148-021-01142-1 (PMC8360816; doi:10.1186/s13148-021-01142-1)
Supplement: Supplementary file 1 — Additional file 1. Supplementary tables and figures. [file 13148_2021_1142_MOESM1_ESM.docx]

**ADDITIONAL FILE FIGURES**


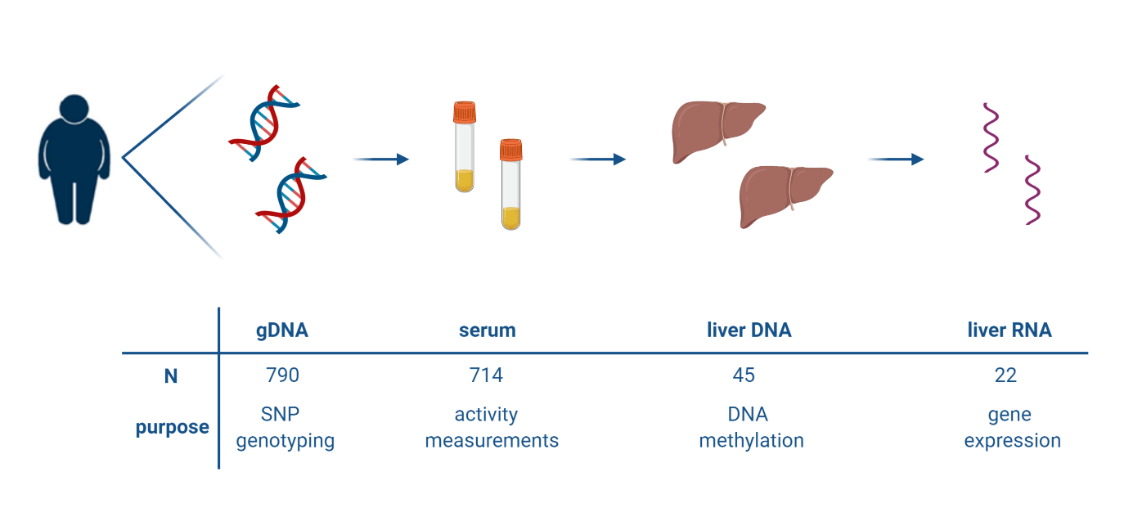


**Additional file 1: Figure S1. Schematic overview of the study population.** The HEPADIP cohort consists of individuals affected by obesity with or without the presence of non-alcoholic fatty liver disease. HEPADIP subcohorts are created based on the different levels of PON1 variability (genetics, epigenetics, expression, and activity). Sample types are presented with corresponding size and purpose.


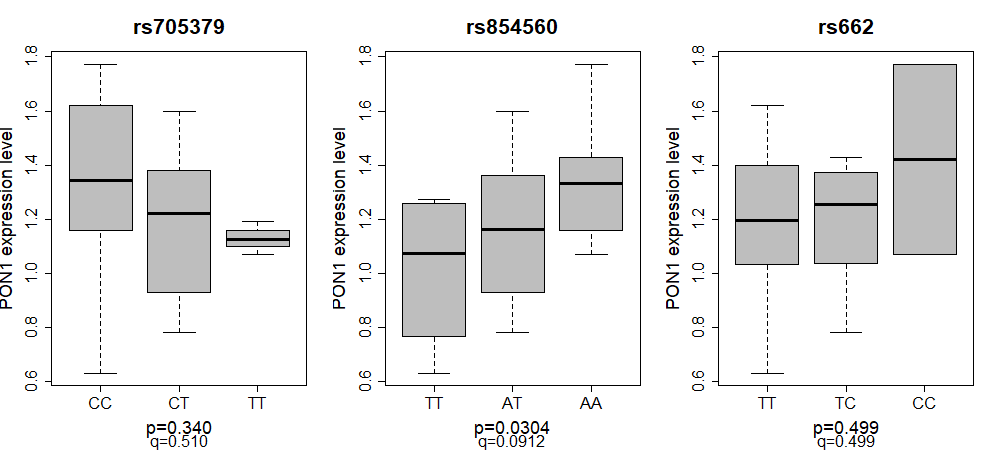


**Additional file 1: Figure S2. Genetic effect of *PON1* on gene expression.** The different boxplots indicate the relationship between each genotype of the three common polymorphisms rs705379:C>T, rs854560:A>T, rs662:T>C on relative *PON1* expression levels in a population of patients with a wide range of (hepato)metabolic derangements. A total of 22 RNA samples were analysed for which genotype distribution over the three PON1 variants is as follows: 32% CC, 54% CT and 14% TT for rs705379:C>T; 18% TT, 41% AT and 41% AA for rs854560:A>T; and 50% TT, 41% CT and 9% CC for rs662:T>C. The significance level (p) and FDR threshold (q) were set at 0.05.


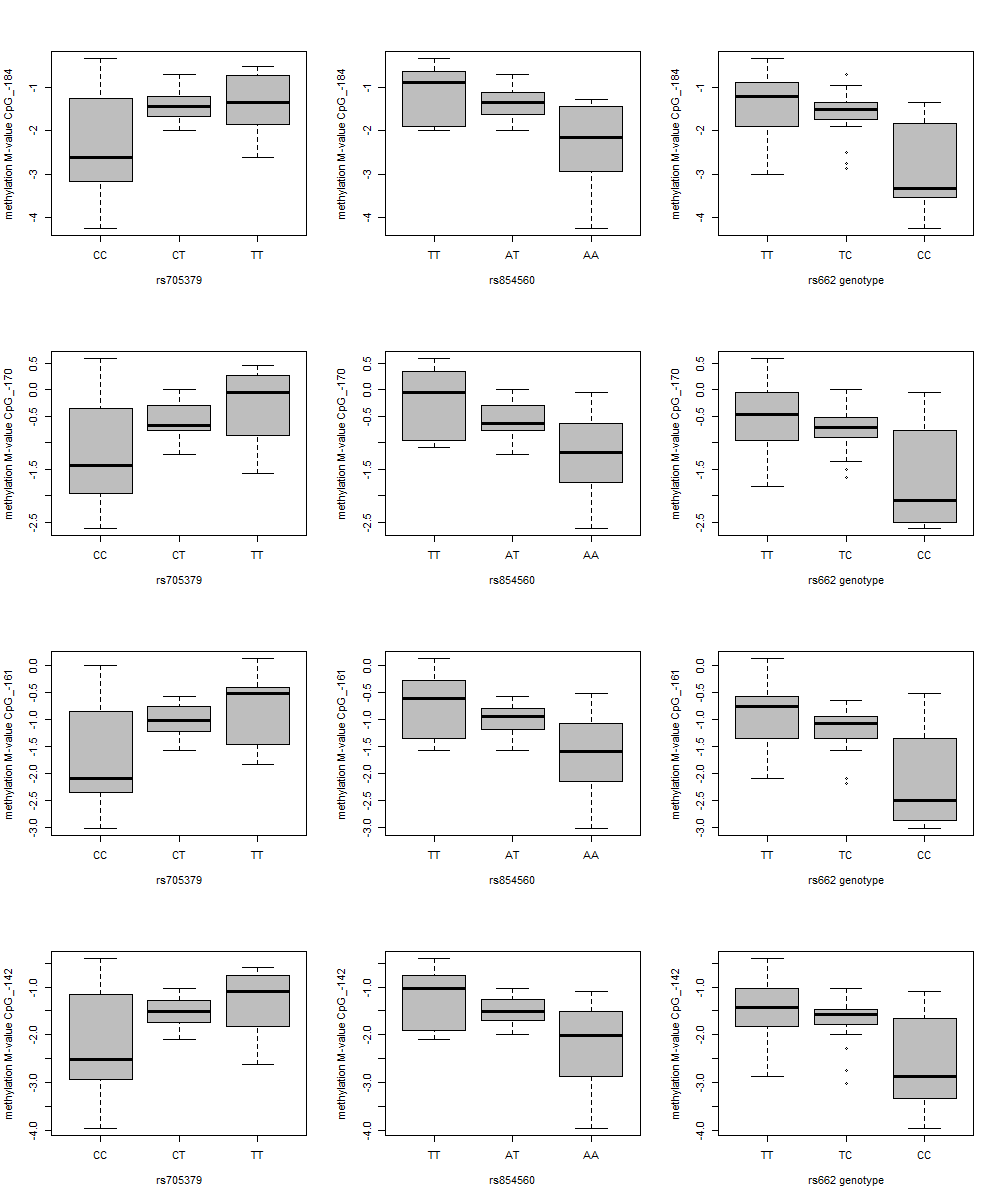


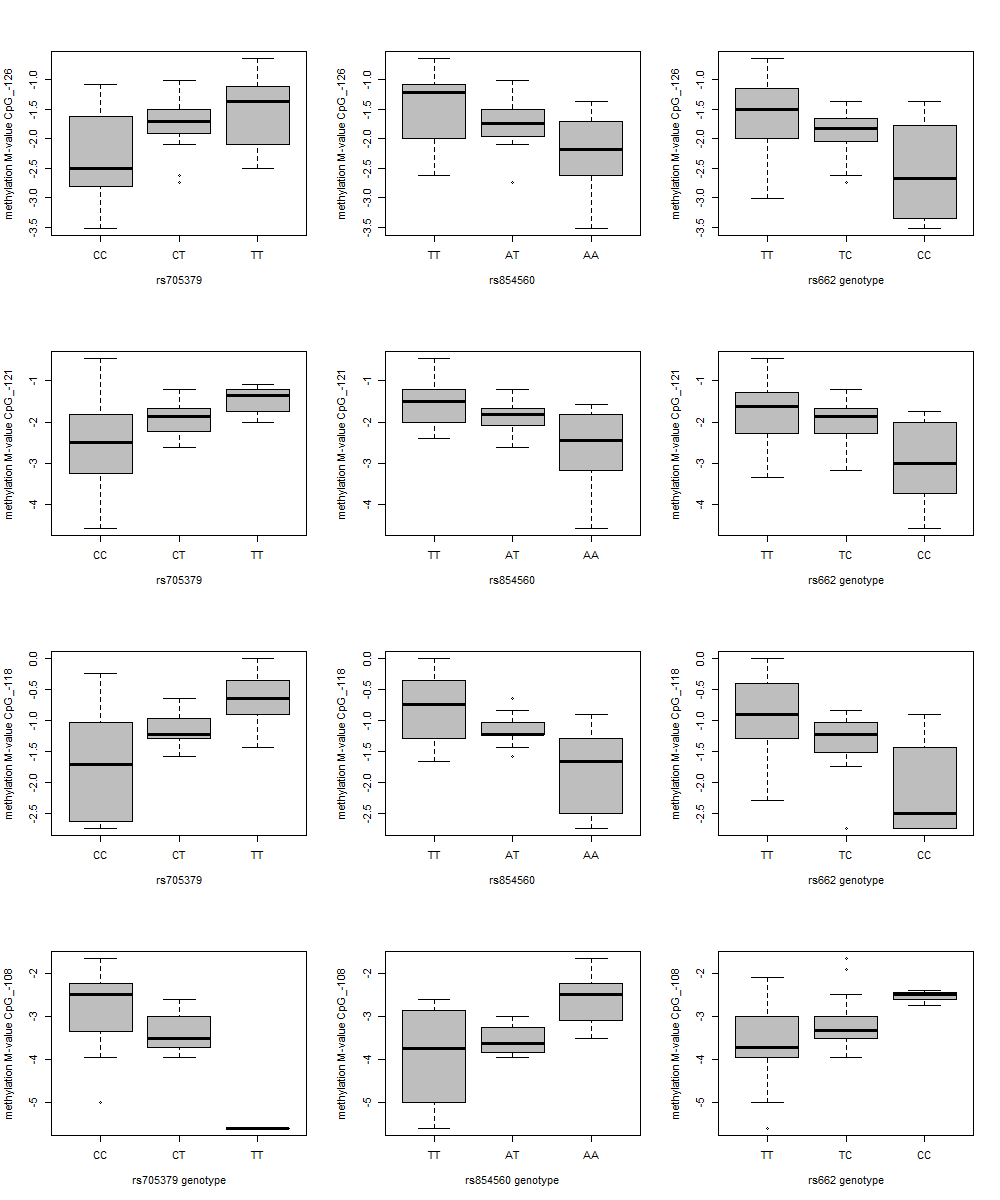


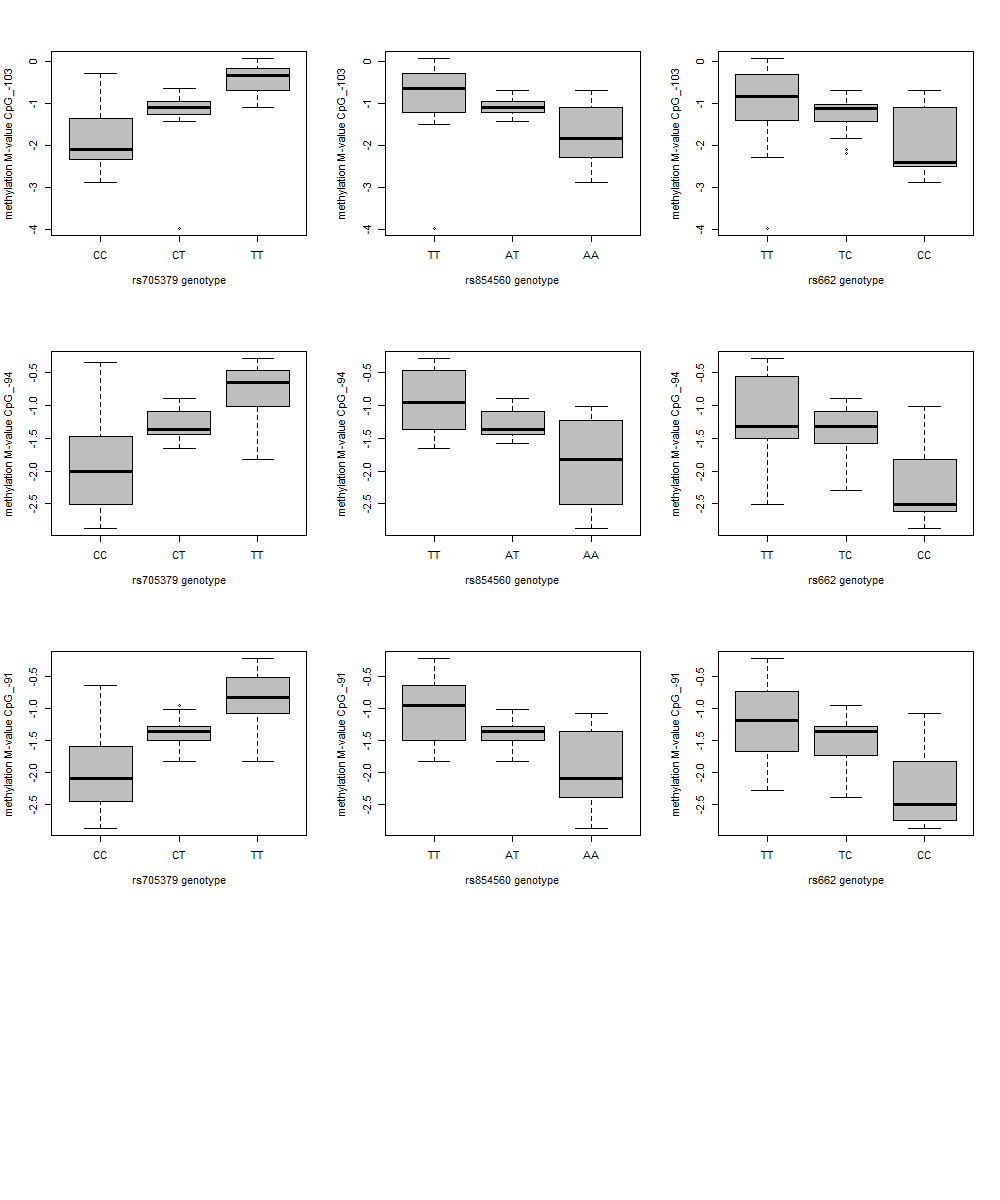


**Additional file 1: Figure S3. Methylation patterns across 11 distinct CpG sites of the *PON1* promoter region in relation to genetics.** Comparison analysis indicates the same genetic effect for all CpG sites, except CpG -*108*. Methylation is represented as methylation values (M-values); positive M-values mean that more molecules are methylated than unmethylated (> 50% methylation) while negative M-values mean the opposite (< 50% methylation).

**
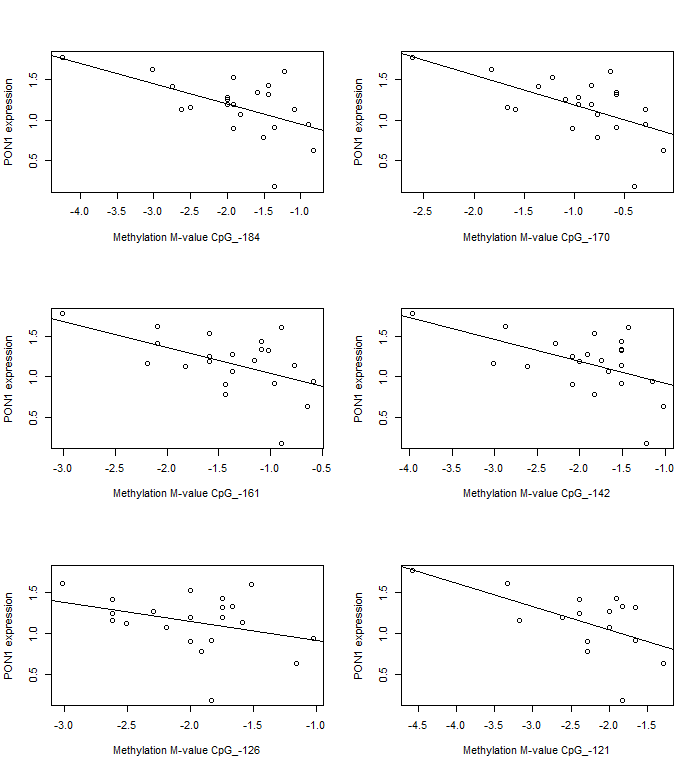
**

**
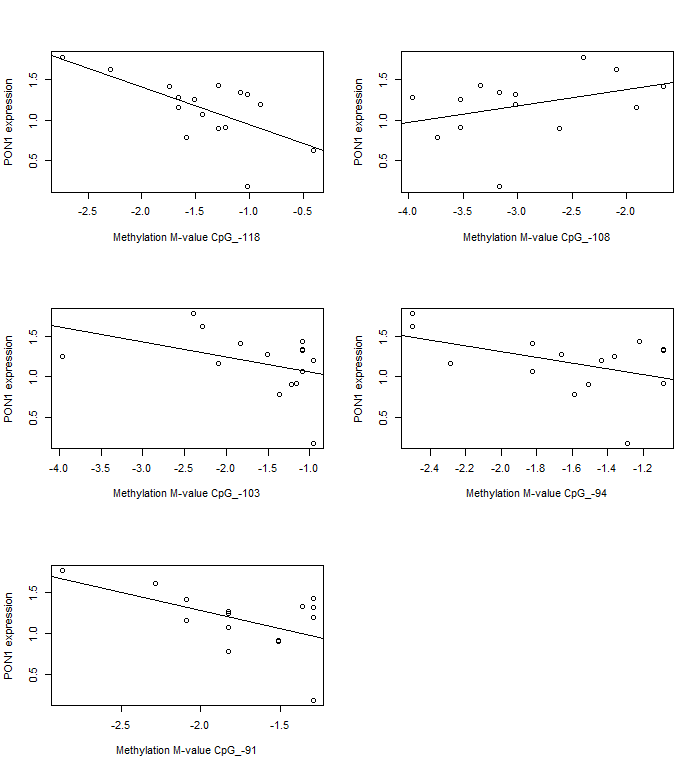
**

**
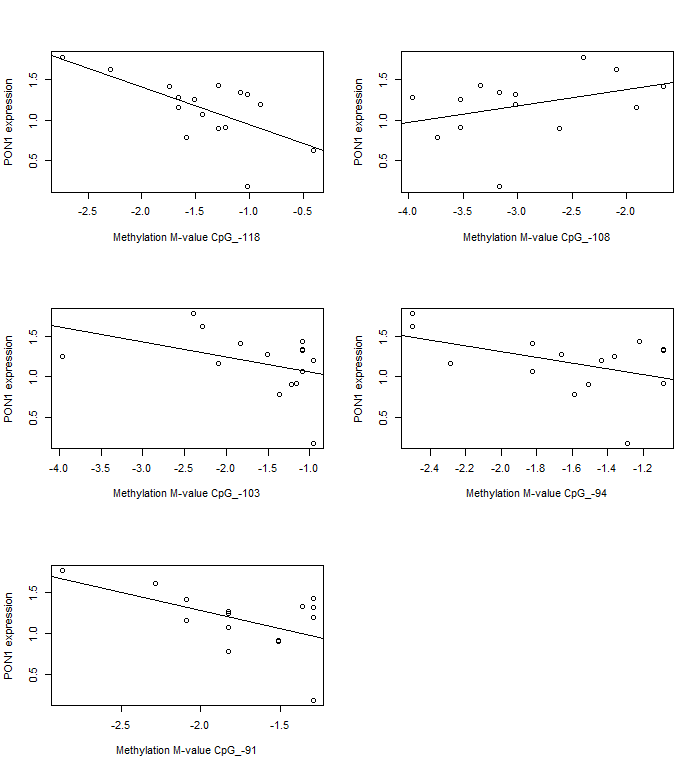
**

**Additional file 1: Figure S4. Methylation patterns across 11 distinct CpG sites of the *PON1* promoter region in relation to expression.** Comparison analysis indicates the same effect for all CpG sites, except CpG -*108*, on relative *PON1* expression. Methylation is represented as methylation values (M-values); positive M-values mean that more molecules are methylated than unmethylated (> 50% methylation) while negative M-values mean the opposite (< 50% methylation).

**
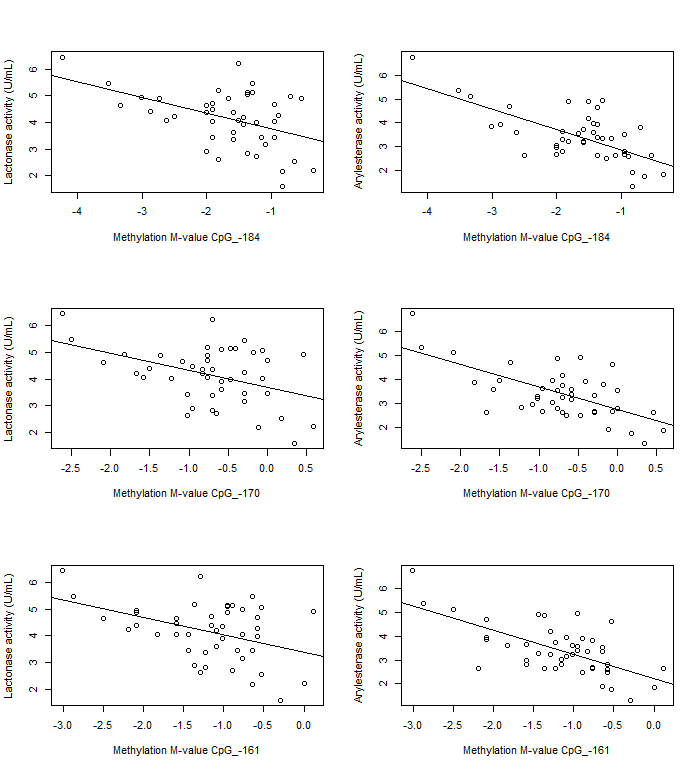
**

**
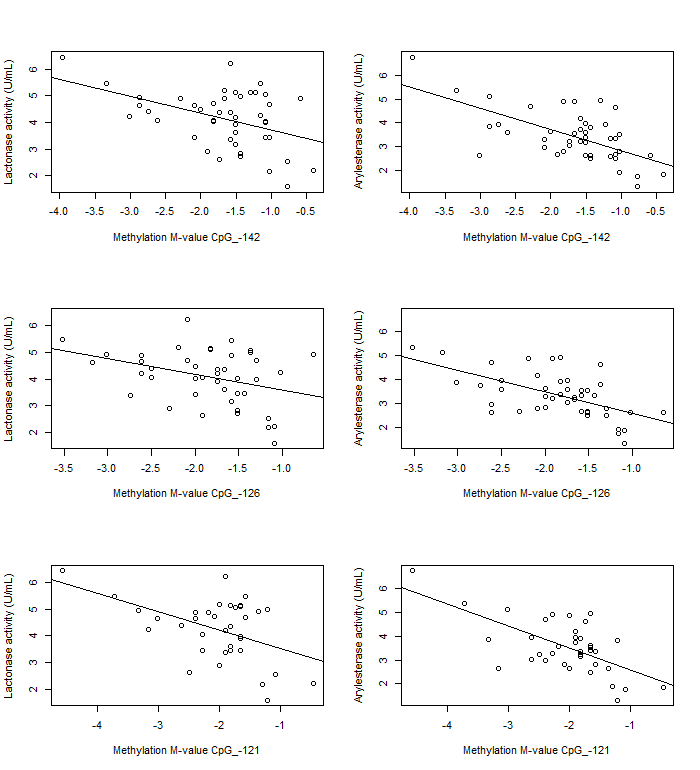
**

**
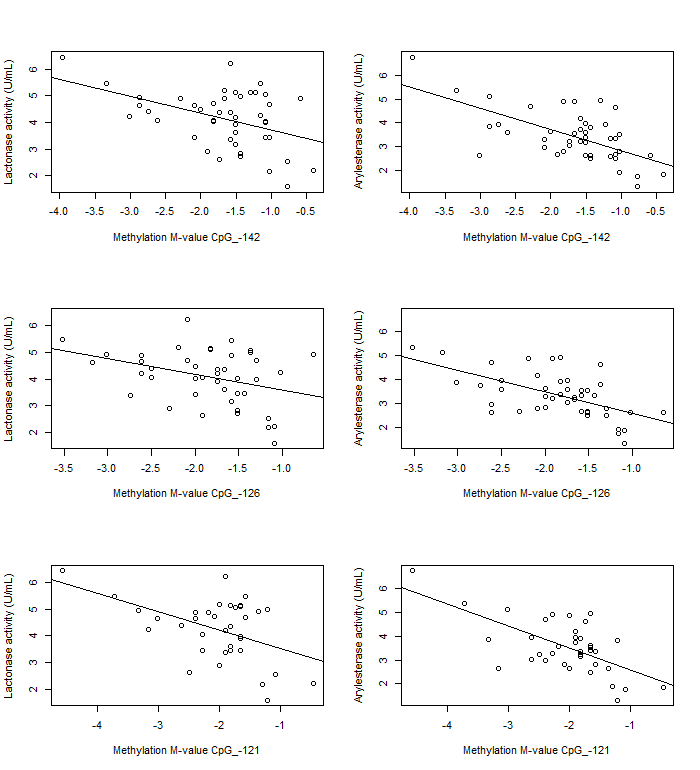
**

**
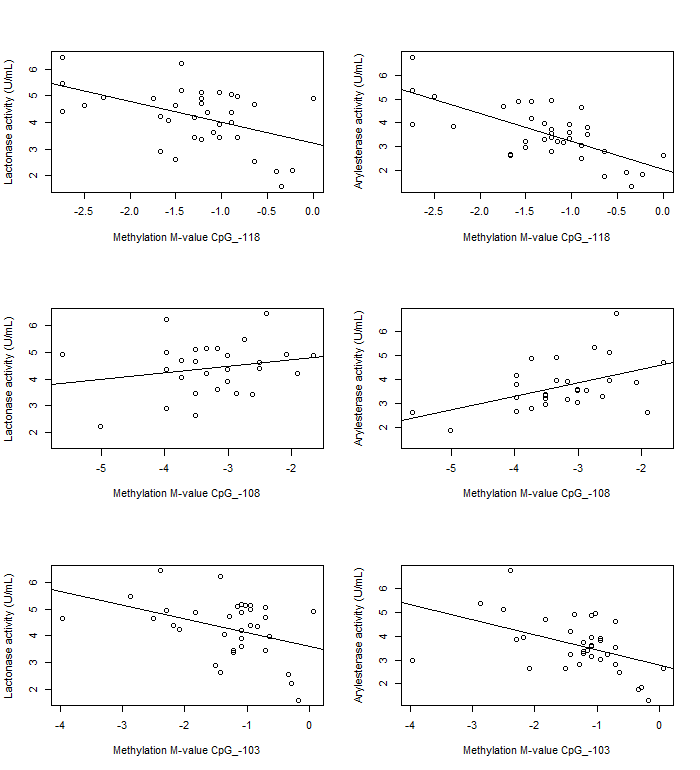
**

**
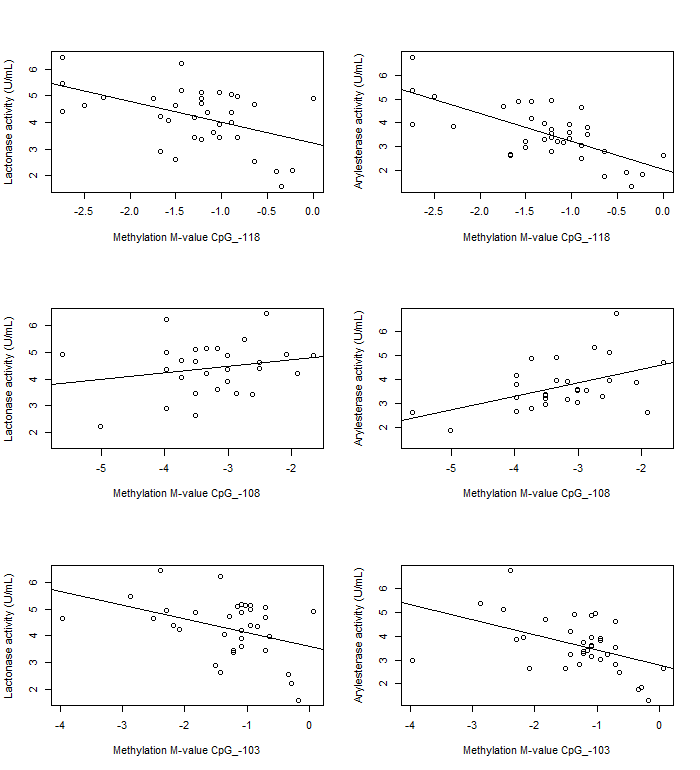
**

**
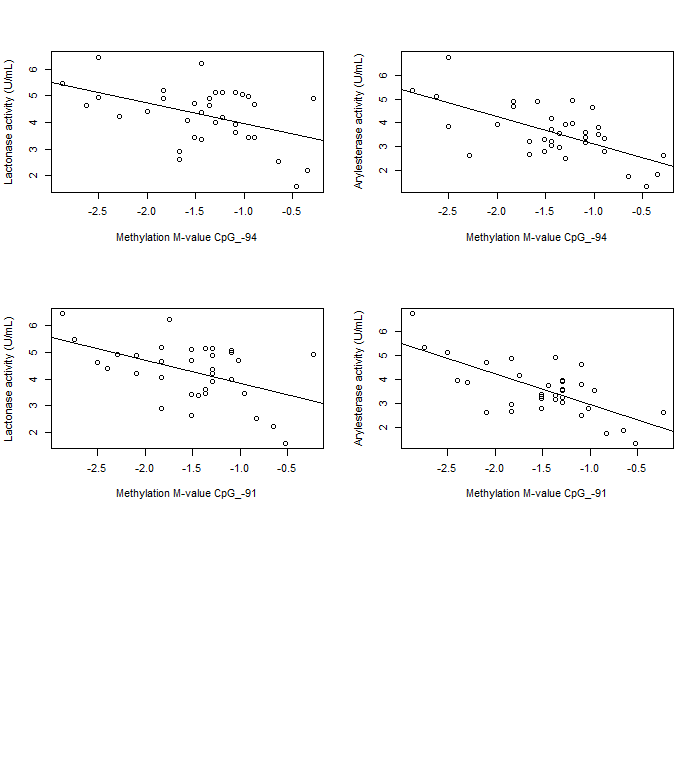
**

**Additional file 1: Figure S5. Methylation patterns across 11 distinct CpG sites of the *PON1* promoter region in relation to substrate-specific enzymatic activity.** Comparison analysis indicates the same effect for all CpG sites, except CpG -*108*, on both enzymatic activities. Methylation is represented as methylation values (M-values); positive M-values mean that more molecules are methylated than unmethylated (> 50% methylation) while negative M-values mean the opposite (< 50% methylation). Activity levels are expressed as units per milliliter of serum, in which 1 unit equals 1 mmol of TBBL (lactone-hydrolyzing activity) or phenyl acetate (arylester-hydrolysing activity) hydrolyzed/min.


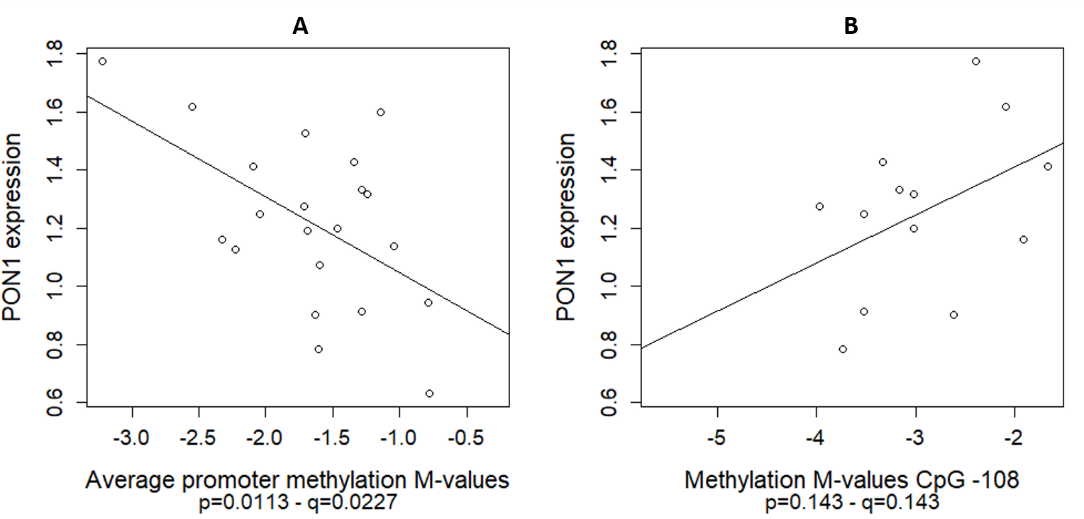


**Additional file 1: Figure S6. Relationship between *PON1* epigenetics and gene expression.** Graphs indicating the association of average promoter methylation (A) and -*108* methylation (B) with relative *PON1* expression levels in a population of patients with a wide range of (hepato)metabolic derangements. Methylation is represented as M-values; positive M-values mean that more molecules are methylated than unmethylated (> 50% methylation) while negative M-values mean the opposite (< 50% methylation). The significance level (p) and FDR threshold (q) were set at 0.05.


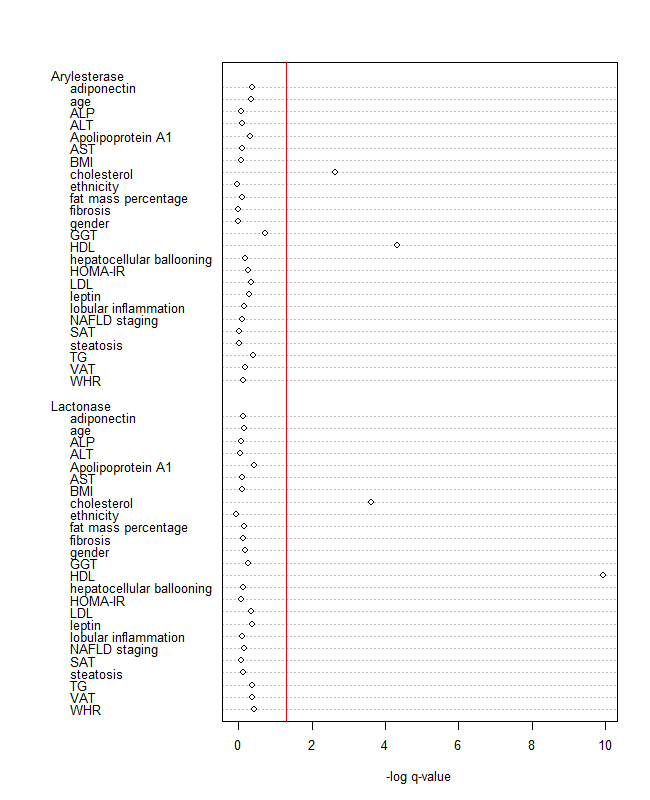


**Additional file 1: Figure S7. Correlation between PON1 status and the hepatometabolic phenotype.** Dot chart indicating the association of PON1 activities arylesterase and lactonase with different phenotypic variables. The –log q-value was used as threshold to define significance (q<0.05) and is displayed as a vertical red line. Abbreviations: ALT = alanine aminotransferase; ALP = alkaline phosphatase; AST = aspartate aminotransferase; BMI = body mass index; GGT = gamma-glutamyltransferase; HDL = ; high-density lipoprotein; HOMA-IR = homeostasis model insulin resistance; SAT = subcutaneous abdominal adipose tissue; TG = triglycerides; VAT = visceral abdominal tissue; WHR = waist-to-hip ratio.

**Additional file 1: Table S1. Enzymatic activity subpopulation characteristics.**

|  | **mean** | **(minimum/maximum)** |
| --- | --- | --- |
| **age (years)** | 42.8 ± 12.7 | (18-74) |
| **weight (kg)** | 110.6 ± 21.5 | (65.8-226.6) |
| **height (m)** | 1.7 ± 0.09 | (1.47-2.06) |
| **BMI (kg/m²)** | 38.8 ± 6.3 | (24.9-69.1) |
| **waist circumference (cm)** | 116.7 ± 14.3 | (83.5-193) |
| **hip circumference (cm)** | 122.2 ± 10.4 | (95-160) |
| **waist-to-hip ratio** | 0.96 ± 0.1 | (0.66-1.4) |
| **fat free mass (kg)** | 56.2 ± 12.2 | (36.5-110) |
| **fat mass (kg)** | 54.3 ± 15.1 | (16.9-134.5) |
| **fat mass (%)** | 48.9 ± 7.6 | (22.3-65.3) |
| **total abdominal adipose tissue (cm²)** | 799.5 ± 185.1 | (246-1386) |
| **visceral abdominal tissue (cm²)** | 195.9 ± 89.3 | (29-567) |
| **subcutaneous abdominal tissue (cm²)** | 603.6 ± 157.8 | (166-1059) |
| **systolic blood pressure (mmHg)** | 127.1 ± 14.5 | (90-180) |
| **diastolic blood pressure (mmHg)** | 75.45 ± 10.3 | (49-115) |
| **creatinine kinase (mg/dL)** | 0.81 ± 0.2 | (0.38-1.16) |
| **aspartate aminotransferase (U/L)** | 23.6 ± 13.4 | (7-133) |
| **alanine aminotransferase (U/L)** | 35.8 ± 23.2 | (7-265) |
| **alkaline phosphatase (U/L)** | 79.9 ± 22.1 | (30-236.03) |
| **gamma-glutamyltransferase (U/L)** | 43.4 ± 33.5 | (11.8-315.2) |
| **total cholesterol (mg/dL)** | 199 ± 39.3 | (75-400.4) |
| **high-density-lipoprotein cholesterol (mg/dL)** | 50.8 ± 13.9 | (24-107.17) |
| **triglycerides (mg/dL)** | 152.9 ± 82.3 | (17-823) |
| **low-density-lipoprotein cholesterol (mg/dL)** | 117.8 ± 34.8 | (17.6-115.6) |
| **insulin resistance (HOMA)** | 18.0 ± 16.1 | (5.4-147.7) |
| **steatosis (0:1:2:3)** | 109:151:119:82 | |
| **ballooning (0:1:2)** | 139:187:135 | |
| **lobular inflammation (0:1:2:3)** | 148:208:75:30 | |
| **fibrosis stage (0:1:2:3:4)** | 287:91:51:28:3 | |
| **Adjusted SAF activity (0:1:2:3:4:5)** | 0:61:112:118:44:16 | |
| **NAFLD activity score (0:1:2:3:4:5:6:7:8)** | 75:44:48:62:84:60:50:30:8 | |
| **NAFLD staging (1:2:3:4)** | 61:80:210:53 | |

Patient clinical and biochemical variable statistics are represented for the enzymatic activity HEPADIP subcohort (N=714; 512 females – 202 males). Values are expressed as the mean ± standard deviation. Lower and upper limits are indicated as minimum/maximum. The distribution of patients (x:y:z) according to the histological characteristics are shown as absolute values. The histological criteria were evaluated by the NASH-CRN scoring system.

**Additional file 1: Table S2. Methylation subpopulation characteristics.**

|  | **mean** | **(minimum/maximum)** |
| --- | --- | --- |
| **age (years)** | 45.6 ± 12.6 | (20-71) |
| **weight (kg)** | 111.6 ± 23.9 | (70.4-174.8) |
| **height (m)** | 1.7 ± 0.1 | (1.54-1.97) |
| **BMI (kg/m²)** | 38.2 ± 5.8 | (24.97-51.0) |
| **waist circumference (cm)** | 117.9 ± 15.2 | (83.5-193) |
| **hip circumference (cm)** | 119.9 ± 10.7 | (97-142) |
| **waist-to-hip ratio** | 0.96 ± 0.1 | (0.71-1.24) |
| **fat free mass (kg)** | 58.0 ± 13.2 | (38.9 - 89.1) |
| **fat mass (kg)** | 52.1 ± 14.9 | (16.9-87) |
| **fat mass (%)** | 47.0 ± 8.4 | (22.3-64.2) |
| **total abdominal adipose tissue (cm²)** | 781 ± 196.4 | (339-1141) |
| **visceral abdominal tissue (cm²)** | 208.7 ± 96.9 | (60-476) |
| **subcutaneous abdominal tissue (cm²)** | 572.3 ± 159.9 | (253-906) |
| **systolic blood pressure (mmHg)** | 127.8 ± 13.4 | (98-152) |
| **diastolic blood pressure (mmHg)** | 76.3 ± 10.3 | (60-97) |
| **creatinine kinase (mg/dL)** | 0.8 ± 0.2 | (0.49-1.2) |
| **aspartate aminotransferase (U/L)** | 31.0 ± 23.2 | (11-133) |
| **alanine aminotransferase (U/L)** | 50.4 ± 43.4 | (9.8-265) |
| **alkaline phosphatase (U/L)** | 79.5 ± 29.0 | (44-187) |
| **gamma-glutamyltransferase (U/L)** | 43.4 ± 33.5 | (18.2-143) |
| **total cholesterol (mg/dL)** | 199 ± 39.3 | (72-119.0) |
| **high-density-lipoprotein cholesterol (mg/dL)** | 48 ± 11.8 | (29.5-72) |
| **triglycerides (mg/dL)** | 152.9 ± 82.3 | (66-321) |
| **low-density-lipoprotein cholesterol (mg/dL)** | 122 ± 32.2 | (51.2-212.2) |
| **insulin resistance (HOMA)** | 22.3 ± 23.2 | (5.4-161.6) |
| **steatosis (0:1:2:3)** | 9:13:14:9 | |
| **ballooning (0:1:2)** | 17:19:09 | |
| **lobular inflammation (0:1:2:3)** | 15:21:6:3 | |
| **fibrosis stage (0:1:2:3:4)** | 25:8:7:4:1 | |
| **Adjusted SAF activity (0:1:2:3:4:5)** | 12:10:12:10:5:2 | |
| **NAFLD activity score (0:1:2:3:4:5:6:7:8)** | 11:9:11:9:1:1:0:0:0 | |
| **NAFLD staging (1:2:3:4)** | 9:12:12:12 | |

Patient clinical and biochemical variable statistics are represented for the methylation HEPADIP subcohort (N=45; 28 females – 17 males). Values are expressed as the mean ± standard deviation. Lower and upper limits are indicated as minimum/maximum. The distribution of patients (x:y:z) according to the histological characteristics are shown as absolute values. The histological criteria were evaluated by the NASH-CRN scoring system.

**Additional file 1: Table S3. Gene expression subpopulation characteristics.**

|  | **mean** | **(minimum/maximum)** |
| --- | --- | --- |
| **age (years)** | 42.3 ± 13.4 | (20-63) |
| **weight (kg)** | 107.3 ± 20.9 | (76.8-161.8) |
| **height (m)** | 1.7 ± 0.1 | (1.57-1.93) |
| **BMI (kg/m²)** | 37.5 ± 5.1 | (27.2-47.5) |
| **waist circumference (cm)** | 114.8 ± 14.1 | (88-137.5) |
| **hip circumference (cm)** | 121.1 ± 9.6 | (108-142) |
| **waist-to-hip ratio** | 0.95 ± 0.1 | (0.71-1.1) |
| **fat free mass (kg)** | 55.4 ± 10.1 | (41.9 - 79.1) |
| **fat mass (kg)** | 51.8 ± 14.2 | (31.2-87) |
| **fat mass (%)** | 47.9 ± 6.9 | (30.4-62.3) |
| **total abdominal adipose tissue (cm²)** | 773 ± 170 | (424-1064) |
| **visceral abdominal tissue (cm²)** | 197.1 ± 89.7 | (60-369) |
| **subcutaneous abdominal tissue (cm²)** | 576.6 ± 152.6 | (298-906) |
| **systolic blood pressure (mmHg)** | 127.2 ± 13.7 | (105-152) |
| **diastolic blood pressure (mmHg)** | 76.5 ± 8.5 | (60-97) |
| **creatinine kinase (mg/dL)** | 0.8 ± 0.1 | (0.5-1) |
| **aspartate aminotransferase (U/L)** | 29.7 ± 19.4 | (11-102) |
| **alanine aminotransferase (U/L)** | 53.1 ± 53.62 | (12-265) |
| **alkaline phosphatase (U/L)** | 74.2 ± 29.0 | (44-64.5) |
| **gamma-glutamyltransferase (U/L)** | 64.5 ± 43.5 | (21.9-143) |
| **total cholesterol (mg/dL)** | 202.2 ± 41.5 | (119-286) |
| **high-density-lipoprotein cholesterol (mg/dL)** | 48 ± 10.6 | (30-65) |
| **triglycerides (mg/dL)** | 149.2 ± 61.1 | (66-321) |
| **low-density-lipoprotein cholesterol (mg/dL)** | 124 ± 37.5 | (51.2-212.2) |
| **insulin resistance (HOMA)** | 24.9 ± 32.27 | (5.4-74.4) |
| **steatosis (0:1:2:3)** | 4:6:6:6 | |
| **ballooning (0:1:2)** | 10:7:5 | |
| **lobular inflammation (0:1:2:3)** | 7:8:6:1 | |
| **fibrosis stage (0:1:2:3:4)** | 11:5:4:1:1 | |
| **Adjusted SAF activity (0:1:2:3:4:5)** | 6:4:4:5:2:1 | |
| **NAFLD activity score (0:1:2:3:4:5:6:7:8)** | 4:2:2:3:3:2:3:3:0 | |
| **NAFLD staging (1:2:3:4)** | 4:7:5:6 | |

Patient clinical and biochemical variable statistics are represented for the gene expression HEPADIP subcohort (N=22; 12 females – 6 males). Values are expressed as the mean ± standard deviation. Lower and upper limits are indicated as minimum/maximum. The distribution of patients (x:y:z) according to the histological characteristics are shown as absolute values. The histological criteria were evaluated by the NASH-CRN scoring system.

**Additional file 1: Table S4. Statistical significance values *PON1* genetics in relation to activity.**

1. **
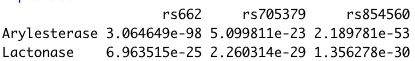
**
2.
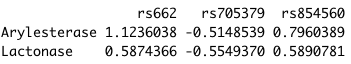


(a) p-values and (b) effect estimate (EE) for each PON1 genetic versus activity pairwise association.

**Additional file 1: Table S5. Statistical significance values *PON1* genetics in relation to methylation.**

1. **
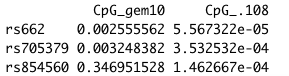
**
2. **
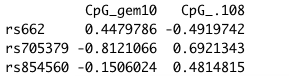
**

(a) p-values and (b) effect estimate (EE) for each PON1 genetic versus methylation pairwise association.
